# Genotype × Isolate Interactions for Tan Spot Resistance Screening in Mungbean

**DOI:** 10.3390/plants15203148

**Published:** 2026-10-15

**Authors:** Krishna Chaitanya Alamuru, Noel L. Knight, Prue Bottomley, Merrill Ryan, Levente Kiss, Anke Martin

**Affiliations:** 1 Centre for Crop Health, School of Science, Engineering and Digital Technologies, University of Southern Queensland, Toowoomba, QLD 4350, Australia; noel.knight@unisq.edu.au (N.L.K.); pruebottomley@hotmail.com (P.B.); levente.kiss@unisq.edu.au (L.K.); 2 Metagen Pty Ltd., Gatton, QLD 4343, Australia; 3 Queensland Department of Primary Industries, Warwick, QLD 4370, Australia; merrill.ryan@dpi.qld.gov.au

**Keywords:** mungbean, bacterial tan spot, *Curtobacterium flaccumfaciens* pv. *flaccumfaciens*, genotype × isolate interaction, disease severity, germplasm screening, pathogen diversity, resistance screening

## Abstract

Tan spot, caused by *Curtobacterium flaccumfaciens* pv. *flaccumfaciens* (Cff), is an important bacterial disease of mungbean (*Vigna radiata*) in Australia. A key question for resistance screening is whether the relative resistance or susceptibility of mungbean genotypes depends on the isolate used. We evaluated ten Australian Cff isolates in two studies comprising 30 diverse mungbean genotypes (Study 1) and 12 breeding lines and varieties (Study 2), with two glasshouse trials per study. Primary analyses excluded the non-pathogenic control Cff089. Host genotype, isolate and genotype × isolate interaction affected disease severity in both studies (*p* < 0.001). Isolate and genotype × isolate interaction accounted for 17.3% and 25.3% of total variance in Study 1, and 17.1% and 53.1% in Study 2, respectively. The rank order of the nine pathogenic isolates was not consistent between studies; only two retained the same rank (Spearman ρ = −0.08, *p* = 0.83). Because the genotype panels did not overlap, this cross-study ranking comparison is descriptive. Genotype AGG325972MUNG had the lowest estimated disease severity in Study 1 (62.1) and showed low mean severity across the eight pathogenic isolates for which observations were available. These results show that isolate choice can alter the apparent resistance of germplasm and therefore influence selection decisions. Screening with multiple characterised Cff isolates should provide a more reliable basis for selecting mungbean material for tan spot resistance.

## 1. Introduction

Tan spot, caused by *Curtobacterium flaccumfaciens* pv. *flaccumfaciens* (Cff), is an important bacterial disease of mungbean (*Vigna radiata* (L.) R. Wilczek) in Australia [1,2]. The Australian mungbean industry is valued at approximately A$180 to A$200 million annually, and tan spot resistance is a Tier 1 (high-impact) breeding priority for the National Mungbean Improvement Program [3]. This bacterium is xylem-inhabiting and can enter plants through contaminated seed, wounds and natural openings before colonising vascular tissue [4]. Infection produces interveinal chlorosis that progresses to pale brown or tan necrotic lesions, and wilting can follow as vascular function declines [1,4]. Tan spot was first reported on Australian mungbean in Queensland in 1984 and in New South Wales in 1986 [2]. Management relies mainly on disease-free seed, removal of alternative hosts and infested residues, and crop rotation [4]. However, Diatloff and Imrie [5] found that clean seed schemes had not sufficiently reduced tan spot incidence in Queensland and concluded that genetic resistance was the most practical management option.

Australian Cff isolates are genetically diverse. Whole-genome analysis of isolates collected between 1986 and 2019 resolved six genetic clusters and showed that a clonal lineage present in the 1980s has persisted and expanded in Australian mungbean [6]. Isolates also differ in plasmid number and in published copy numbers of pCff119, a plasmid investigated in relation to pathogenicity [2,7]. Isolate Cff089, in which no plasmid was detected, has previously been characterised as non-pathogenic on mungbean [6,7].

For resistance screening, a genotype × isolate interaction means that the apparent resistance or susceptibility of a genotype can depend on the isolate used. Diatloff and Imrie [5] demonstrated this in Australia by testing eight mungbean varieties against two Cff strains selected from ten to represent contrasting virulence. They found a significant host × strain interaction, showing that cultivar responses depended on the strain used. Beyond mungbean, Cff has also been reported on common bean (*Phaseolus vulgaris*), cowpea (*Vigna unguiculata*) and soybean (*Glycine max*) [1,4]. Valdo et al. [8] identified candidate differential common bean genotypes by testing 30 genotypes against eight Cff isolates. Zia et al. [9] found that the resistance-associated markers detected depended on which of three Cff isolates was used for phenotyping. Together, these studies demonstrate that host responses can be isolate-specific. Recent Australian studies measured disease severity on Jade-AU [6] and Opal-AU [7], respectively, but did not establish whether Australian Cff isolates show consistent disease-severity rankings across different sets of mungbean genotypes. Published tan spot ratings do not identify the isolates used for screening [10]. Historical changes in variety ratings cannot establish increased pathogen aggressiveness because isolate choice and screening methods may also have changed [6,11,12]. Screening with a defined set of characterised isolates would provide clearer context for resistance assessment.

We evaluated ten Australian Cff isolates on two independent sets of host genotypes. Study 1 contained 24 germplasm accessions, two breeding lines and four varieties, while Study 2 contained six advanced breeding lines and six varieties. The ten isolates represented the six published genetic clusters and collections from Queensland and New South Wales between 1986 and 2019 [6]. Both studies used the same inoculation method and disease severity scale, but they shared no common genotypes and were analysed separately. The primary aim was to determine how host genotype, isolate and genotype × isolate interaction affected tan spot severity and whether the rank order of isolates for disease severity was consistent across the two genotype sets. We also aimed to identify genotypes with low disease severity across multiple isolates and replicated genotype × isolate contrasts that could be examined further as candidate differential responses.

## 2. Materials and Methods

### 2.1. Plant Material

Two genotype sets were evaluated in separate studies, referred to hereafter as Study 1 and Study 2.

Study 1 comprised 30 genotypes selected to represent the genetic diversity of mungbean germplasm available to Australian breeding programs (Table 1). These included accessions from the World Vegetable Center mungbean mini core collection, established to capture the diversity of the wider global germplasm collection [13], and from the Australian mungbean diversity panel [14]. The study also included four varieties and two elite pre-release breeding lines (Table 1).

Study 2 comprised six varieties and six advanced breeding lines, giving a total of 12 genotypes (Table 1). These included one black gram (*Vigna mungo*) variety, Onyx-AU; the remaining 11 genotypes were mungbean (*Vigna radiata*).

Seed was obtained from the Australian Grains Genebank (Horsham, Victoria) and the Queensland Department of Primary Industries through the Australian National Mungbean Improvement Program, as detailed in Table 1.

### 2.2. Pathogen Isolates

Ten Cff isolates were selected to represent the known genetic and geographic diversity of the Australian Cff population (Table 2). They were collected in Queensland and New South Wales between 1986 and 2019. Eight isolates originated from mungbean (*Vigna radiata*), DAR 56677 from black gram (*Vigna mungo*), and DAR 58706 from azuki bean (*Vigna angularis*). The set included isolates from all six genetic clusters identified by Sparks et al. [6] and Cff089, which has admixed ancestry between clusters 2 and 5. Isolates collected in 2014, 2015, 2018 and 2019 were sourced from the culture collection held at the Centre for Crop Health, University of Southern Queensland, and are identified by their Queensland Plant Pathology Herbarium (BRIP) accession numbers. The three historical isolates from the 1986–1987 outbreaks (DAR 56672, DAR 56677 and DAR 58706) were obtained from the New South Wales Plant Pathology and Mycology Herbarium (DAR Culture Collection, Orange, New South Wales).

Published droplet digital PCR data were available for nine of the ten isolates. Cff089 was the only isolate in which the pathogenicity-associated linear plasmid was not detected, and it has previously been characterised as non-pathogenic on mungbean [6,7]. Published pCff119 copy numbers are listed in Table 2 [7]. Plasmid detection was not performed in the present study.

All isolates were maintained as glycerol stocks at −80 °C and subcultured on nutrient agar at 27 °C for 48 h before inoculation.

### 2.3. Experimental Design

Each study comprised two glasshouse trials, one in 2023 and one in 2024. Year was included as a fixed effect to account for differences between the two trials within each study. All four trials were conducted at the University of Southern Queensland, Toowoomba, with planting commencing in January of each year. The same ten Cff isolates, inoculation method and disease assessment method were used in both trials.

Each trial used a factorial combination of genotypes and isolates arranged in two randomised complete blocks. Each genotype × isolate combination was represented by one plant in each block, giving two plants per combination per trial. This gave four plants per genotype × isolate combination across the two trials. Analyses included only plants with scores for all three trifoliate leaves (Table S1). Per trial, the design provided for 600 plants in Study 1 (30 genotypes × 10 isolates × 2 blocks) and 240 plants in Study 2 (12 genotypes × 10 isolates × 2 blocks). Three genotypes were grown in each 200 mm diameter pot (4.5 L) filled with Searles Premium Advanced Potting Mix (Searles, Kilcoy, QLD, Australia). Genotypes were randomly assigned to pots. Two seeds per genotype were sown at approximately 2 cm depth and thinned to one plant after emergence. All three plants in a pot received the same isolate. Isolate was therefore applied at the pot level, while genotype varied among the three plants. With three plants per pot, Study 1 comprised 200 pots per trial and Study 2 comprised 80 pots per trial. After emergence, plants were fertilised with Osmocote Tomato, Vegetable & Herb (N:P:K 17:2:7; Scotts, Bella Vista, NSW, Australia) at the manufacturer’s recommended rate. Legume inoculant (Group I, CB1015 *Mesorhizobium jarvisii*; NoduleN peat-based inoculant, New Edge Microbials, Wodonga, VIC, Australia) was applied at planting as a liquid inoculant at the manufacturer’s recommended label rate. The legume inoculant was applied directly to the seed using a pipette. Plants were watered manually at the soil surface until Cff inoculation. After inoculation, pots were sub-irrigated twice weekly by standing the pot bases in approximately 1 cm of water. The glasshouse was maintained under a natural photoperiod at 24–28 °C during the day and 18–22 °C at night.

### 2.4. Inoculum Preparation and Inoculation

Inoculum preparation and stem inoculation used a protocol similar to that described by Saad et al. [7], with the conditions used in the present study detailed below. Bacterial cultures were streaked onto nutrient agar (NA; Amyl Media, Melbourne, Australia) and incubated at 27 °C for 48 h. A single colony was transferred into 10 mL of nutrient broth (NB; Amyl Media) and incubated at 27 °C with shaking at 180 rpm for 48 h using a shaker (Ratek Instruments, Boronia, VIC, Australia). Cultures were centrifuged at 11,200× *g* for 8 min using a centrifuge (Eppendorf, Hamburg, Germany). The supernatant was discarded and the pellet was resuspended in 8 mL of autoclaved Milli-Q ultrapure water (nominal resistivity 18.2 MΩ·cm at 25 °C). Each suspension was adjusted to an OD_600_ of 1.0 with a DS-11FX spectrophotometer (DeNovix, Wilmington, DE, USA), corresponding to approximately 10^8^ colony-forming units (CFU) mL^−1^. The adjusted suspensions were serially diluted and plated to verify the viable bacterial concentration.

Plants were inoculated 14 days after planting, once the first trifoliate had emerged. A 10 μL droplet of inoculum was placed on the stem between the unifoliate leaves and the base of the first trifoliate leaf petiole. A sterile acupuncture needle (0.30 mm diameter; Dongbang Medical, Boryeong, Republic of Korea) was passed through the droplet and stem three times to introduce the bacteria.

### 2.5. Disease Assessment

Disease was assessed 15 days after inoculation. The first three trifoliate leaves of each plant were scored separately. Each leaf received one visual estimate of the percentage of its area showing interveinal chlorosis and/or necrosis, from 0% (no visible symptoms) to 100% (the entire leaf affected). Areas showing both symptoms were counted only once. The three scores were summed to give one plant-level disease severity score from 0 to 300 (Severity_sum = Tri1 + Tri2 + Tri3) [7]. All assessments were made by the same assessor. The summed score quantified disease severity (the extent of symptomatic tissue), rather than disease incidence (the proportion of plants showing symptoms).

Records without any trifoliate scores represented failed germination or late-germinating seedlings removed before inoculation. Missing trifoliates on partially scored plants were generally observed to be associated with plant development or leaf loss rather than disease. Leaves that had dried in response to inoculation were scored as diseased, not treated as missing.

### 2.6. Statistical Analysis

The two studies were analysed separately. Mixed models were fitted in R v4.5.2 [15] using lme4 v1.1-38 [16], with inference and estimated marginal means calculated using lmerTest v3.2-1 [17], emmeans v2.0.4 [18] and pbkrtest v0.5.5 [19]. Clustering, principal component analysis (PCA) and figures were produced in Python v3.12.14 using SciPy v1.18.1, scikit-learn v1.9.1 and Matplotlib v3.11.2 [20,21,22].

Only plants with three recorded trifoliate scores contributed a 0–300 disease-severity score. Incomplete records were excluded; assessed plants without symptoms retained valid zero scores. Plant accounting and recorded scores are provided in Table S1 and Supplementary Data S1, respectively. Primary analyses included the nine pathogenic isolates; Cff089 was retained as a non-pathogenic control in descriptive summaries and all-ten-isolate sensitivity analyses.

### 2.7. Linear Mixed Models and Analysis of Variance

The primary model included genotype, isolate, year and genotype × isolate as fixed effects, with a random intercept for pot nested within year and block to account for dependence among plants sharing a pot [23]:

Severity_sum ~ Genotype + Isolate + Year + Genotype × Isolate + (1 | Year:Block:Pot)

Models were fitted by restricted maximum likelihood with sum-to-zero contrasts and Kenward–Roger inference [19,24,25]. Main effects were tested jointly using equally weighted observed-cell estimated marginal means (EMMs) [26]. The genotype × isolate interaction was tested over its full estimable interaction space, rather than using the observed-cell weighting applied to main effects. Supporting comparisons with Type III tests are provided in Table S2.

### 2.8. Year Effects and the Decision to Combine Trials

Year remained a fixed effect in the primary model. Year interactions were assessed by Kenward–Roger approximate *F* tests comparing nested models (Table S3). Study 2 sensitivity analyses retained genotype × year as a fixed effect and estimated its variance in a separate all-random model (Supplementary Method S3).

### 2.9. Variance Partitioning

A separate model fitted genotype, isolate, genotype × isolate, year and pot as random effects [27]. Each variance component was expressed as a proportion of total variance, including residual variance (Table S4). An all-ten-isolate comparison included Cff089 using the same random-effects structure; year remained fixed in the primary inferential model.

### 2.10. Estimated Marginal Means and Multiple Comparisons

Genotype and isolate EMMs, standard errors and 95% confidence intervals (CI) were calculated with equal weighting of observed genotype × isolate cells. Genotype EMMs averaged responses across isolates and trials; isolate EMMs averaged responses across the genotypes and trials within each study. Pairwise comparisons used Tukey adjustment [28] at *α* = 0.05 and were summarised with compact-letter groupings [29].

### 2.11. Genotype Grouping and Cluster Analysis

Descriptive hierarchical clustering used genotype × isolate cell EMMs, average linkage and Euclidean distances on the unscaled 0–300 scale. Study 1 distances used pairwise-observed combinations without imputation. Group selection and checks of clustering stability [30,31,32,33] are described in Supplementary Method S5. Clusters were not interpreted as pathotypes.

PCA used the nine pathogenic isolates and genotypes with complete isolate profiles, with each isolate centred and scaled to unit variance. A second PCA retained all 30 Study 1 genotypes using a method that accommodates missing observations, to assess whether incomplete profiles affected the patterns (Supplementary Method S5). Within-study isolate response profiles were compared using pairwise-complete Spearman correlations, excluding Cff089. Genotype categories were compared using Kruskal–Wallis tests in Study 1 [34] and Mann–Whitney U tests in Study 2 [35], with genotype as the unit of observation.

### 2.12. Assessment of Between-Trial Agreement

Using the nine pathogenic isolates, models were fitted separately to each trial, omitting year. Genotype and isolate EMMs were compared between trials using Pearson and Spearman correlations; differences in severity and the influence of individual genotypes or isolates were also assessed (Supplementary Method S6).

### 2.13. Descriptive Comparison Across Studies

Pearson and Spearman correlations compared the nine pathogenic isolates’ EMMs between studies. Because the studies shared no genotype, these comparisons were descriptive. Genotype EMMs and 95% confidence intervals from both studies were displayed on the same disease-severity scale, and differences between studies were not statistically tested.

### 2.14. Diagnostic and Sensitivity Analyses

Residual and quantile–quantile plots were used to assess model assumptions. Sensitivity analyses examined the Study 2 genotype × year term, Type III versus joint tests (Table S2), inclusion of Cff089 (Table S5), and incomplete leaf scoring (Table S6). To assess the effect of incomplete leaf scoring, missing scores in partially scored plants were assigned 0 in one sensitivity analysis and 100 in another; recorded scores were unchanged. Assigned values were used only for sensitivity analysis. Supplementary Methods S2, S3 and S7 describe the alternative fixed-effect tests, year-interaction models and Cff089/missing-score analyses, respectively.

## 3. Results

### 3.1. Disease Symptoms

**Figure 1:**
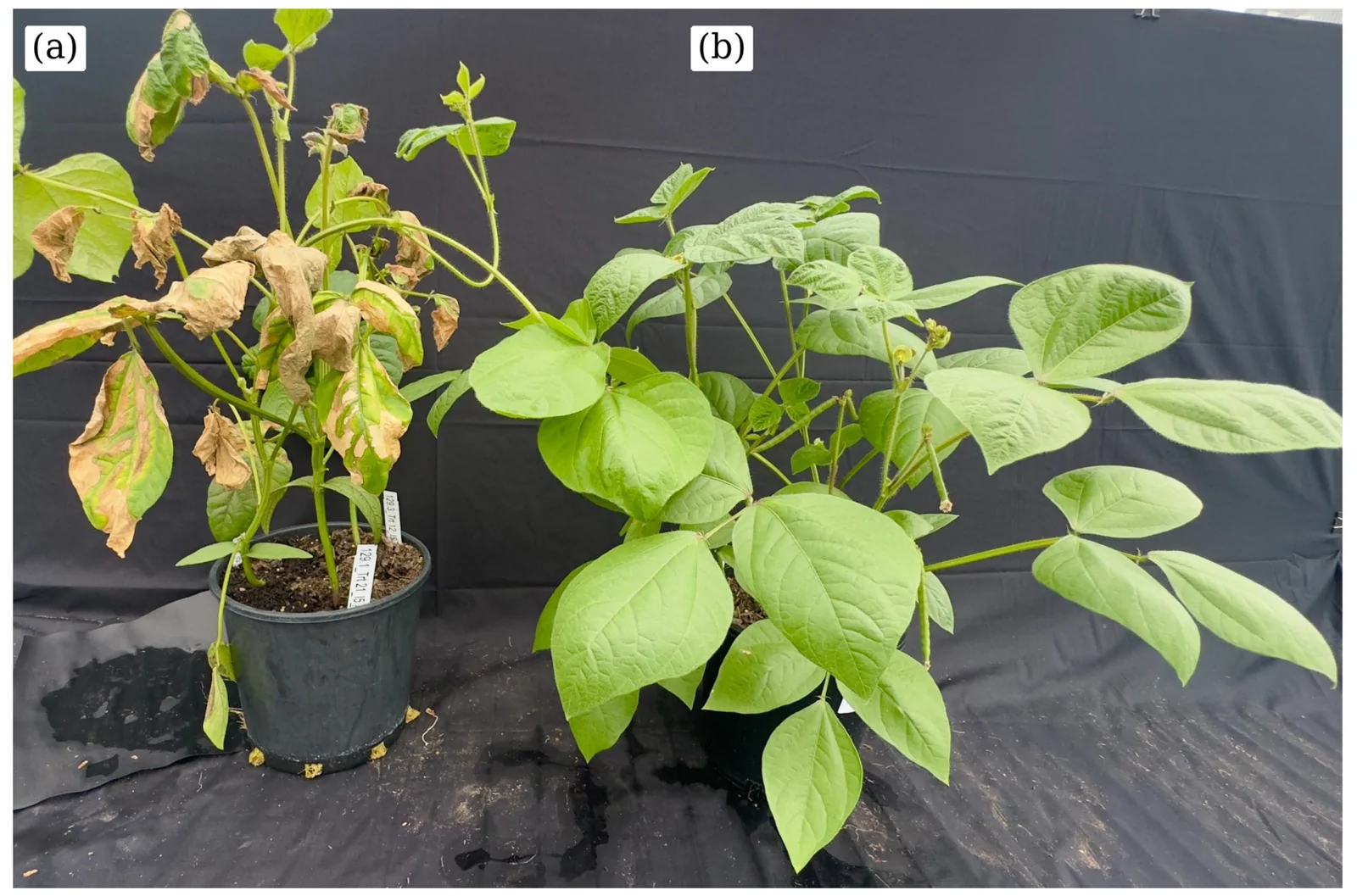
Representative symptoms observed following inoculation with *Curtobacterium flaccumfaciens* pv. *flaccumfaciens* (Cff). Each pot contained three plants of three different genotypes, all inoculated with the same isolate. (**a**) Plants inoculated with Cff062, showing interveinal chlorosis, necrosis, wilting and loss of leaf area characteristic of bacterial tan spot. The labelled plant is the germplasm accession AGG326952MUNG, which had a disease severity score of 275 on the 0–300 scale. (**b**) The corresponding AGG326952MUNG plant inoculated with Cff089, previously characterised as non-pathogenic on mungbean, showed no symptoms and had a disease severity score of 0.

Inoculation with the nine pathogenic Cff isolates produced the characteristic symptoms of bacterial tan spot. Symptoms appeared on trifoliate leaves within three days of inoculation. Interveinal chlorosis progressed to necrosis and pale brown to tan lesions. Severely affected plants developed wilting, brown papery necrotic leaves and substantial loss of leaf area (Figure 1). Disease was assessed 15 days after inoculation, when symptoms were well developed. Only two of the 168 plants inoculated with Cff089 had non-zero disease severity scores; both were in Study 1.

### 3.2. Overall Disease Severity and Its Distribution

**Figure 2:**
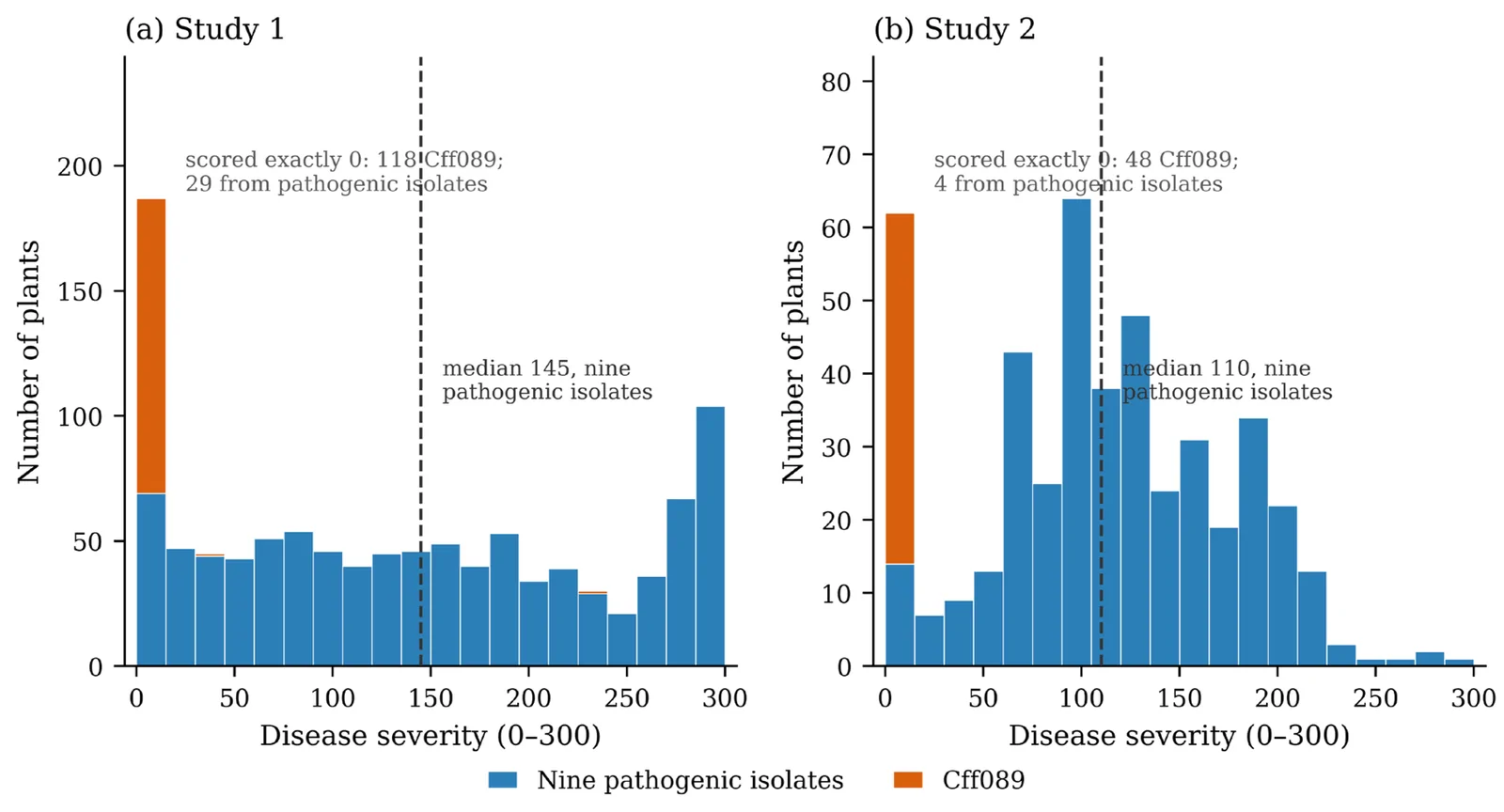
Distribution of disease severity scores across analysed plants in (**a**) Study 1 (*n* = 1077) and (**b**) Study 2 (*n* = 460). The disease severity score is the sum of the three trifoliate ratings on a 0–300 scale, where 0 indicates no disease and 300 indicates maximum disease on all three leaves. Blue bars represent plants inoculated with one of the nine pathogenic *Curtobacterium flaccumfaciens* pv. *flaccumfaciens* (Cff) isolates. Orange bars represent Cff089, previously characterised as non-pathogenic on mungbean. The leftmost class spans scores from 0 to 15, so it includes zero and low non-zero scores. Exact zero counts are printed in each panel. The dashed line shows the median score for plants inoculated with the nine pathogenic isolates. Plants without a complete three-trifoliate score were excluded. Table S1 accounts for all plants sown.

Observed disease severity scores covered the full 0–300 scale in both studies (Figure 2). In Study 1, the mean plant disease severity score was 133 across all ten isolates and 149 across the nine pathogenic isolates. The corresponding medians were 130 and 145. In Study 2, the mean scores were 107 across all ten isolates and 119 across the nine pathogenic isolates, with medians of 110 in both cases.

Cff089 accounted for most zero scores: 118 of 120 plants in Study 1 and all 48 in Study 2. The two non-zero scores, both recorded in Study 1 in 2023, were retained as observed; their cause was undetermined. Zero scores also occurred after inoculation with pathogenic isolates: 29 of 957 Study 1 plants and 4 of 412 Study 2 plants, distributed across 19 genotype × isolate combinations. These were complete assessments of established, inoculated plants that showed no symptoms and were retained as valid observations. Paired-plant observations are summarised in the note to Table S1.

Of 1680 plants sown, 126 were unavailable before inoculation because of failed germination or removal of late-germinating seedlings. Of the remaining 1554 plants, 17 had only one or two trifoliate scores and were excluded from the primary analysis because a complete 0–300 score could not be calculated. Complete assessments were available for 1537 plants. Excluding the 168 Cff089 controls left 1369 plants in the primary analysis (957 in Study 1 and 412 in Study 2). Table S1 provides the full accounting of plants sown and analysed. Under both extreme missing-leaf-score scenarios, genotype, isolate and genotype × isolate remained significant in both studies (all *p* < 0.001; Table S6). In the missing-score sensitivity analyses, genotype rankings shifted by at most one position in Study 1 and two positions in Study 2. Isolate rankings remained unchanged in Study 1 and shifted by at most two positions in Study 2.

### 3.3. Sources of Variation: Genotype, Isolate and Their Interaction

The year main effect was not significant in either study. Among interactions involving year, only genotype × year in Study 2 was significant (*p* = 0.042; Table S3). This interaction explained 1.10% of total variance; retaining it changed genotype EMMs by no more than 0.8 points and did not change genotype or isolate rankings. The two trials were therefore combined for the primary analysis within each study.

In Study 1, genotype, isolate and genotype × isolate interaction all affected disease severity (all *p* < 0.001; Table 3). Variance partitioning attributed 17.3% of total variance to isolate, 25.3% to genotype × isolate interaction and 31.1% to genotype (Table S4). Disease severity therefore reflected both consistent differences among genotypes and changes in genotype response among isolates.

In Study 2, genotype, isolate and genotype × isolate interaction all affected disease severity (all *p* < 0.001; Table 4). Isolate accounted for 17.1% of total variance and genotype × isolate interaction for 53.1%, whereas genotype accounted for 4.4% (Table S4). Thus, most variation in Study 2 was associated with isolate and with changes in genotype response among isolates. The all-ten-isolate variance components are provided for comparison in Table S4. The year variance component was estimated at zero in both studies, with and without Cff089.

### 3.4. Agreement Between Trials

**Figure 3:**
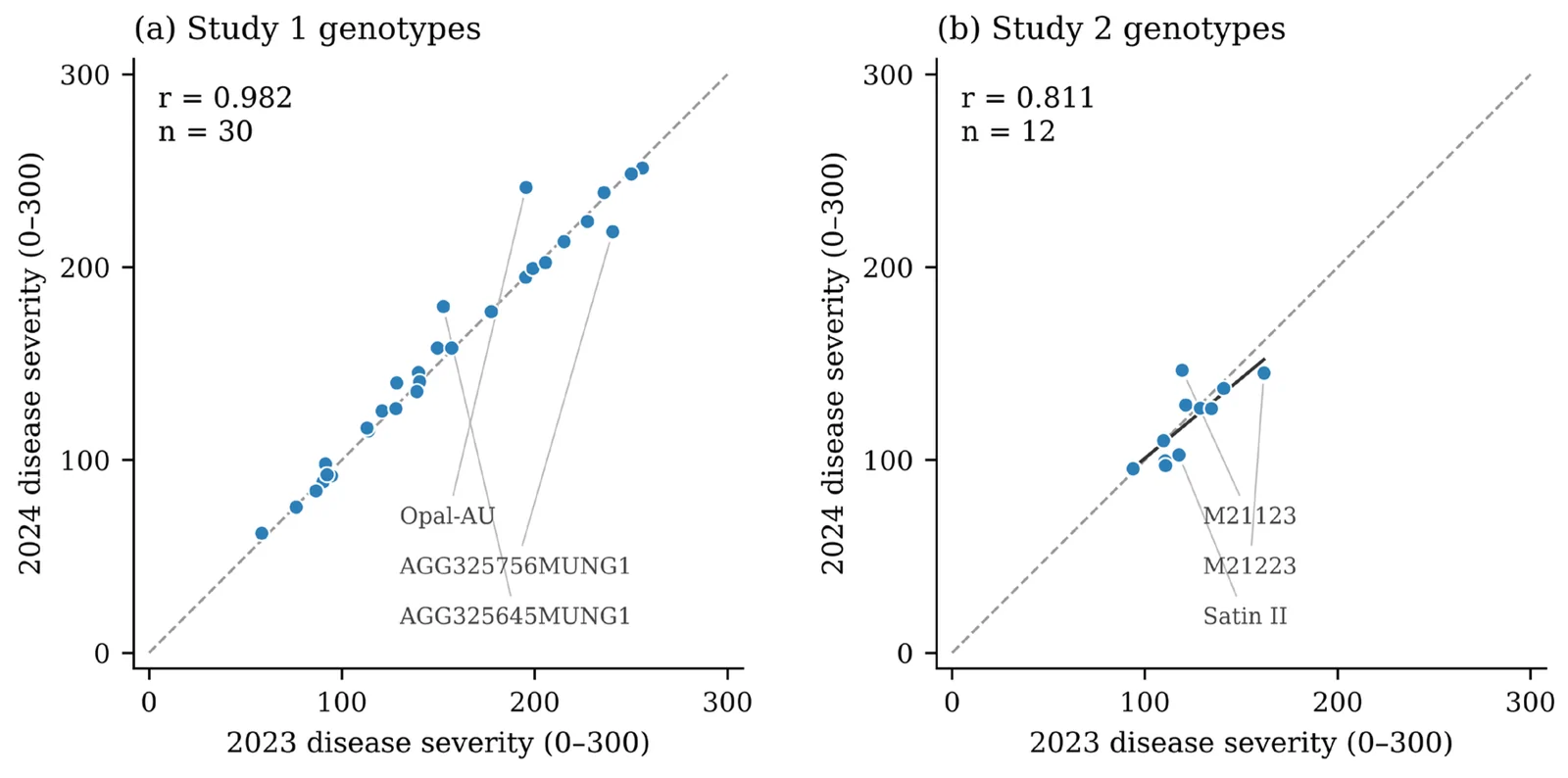

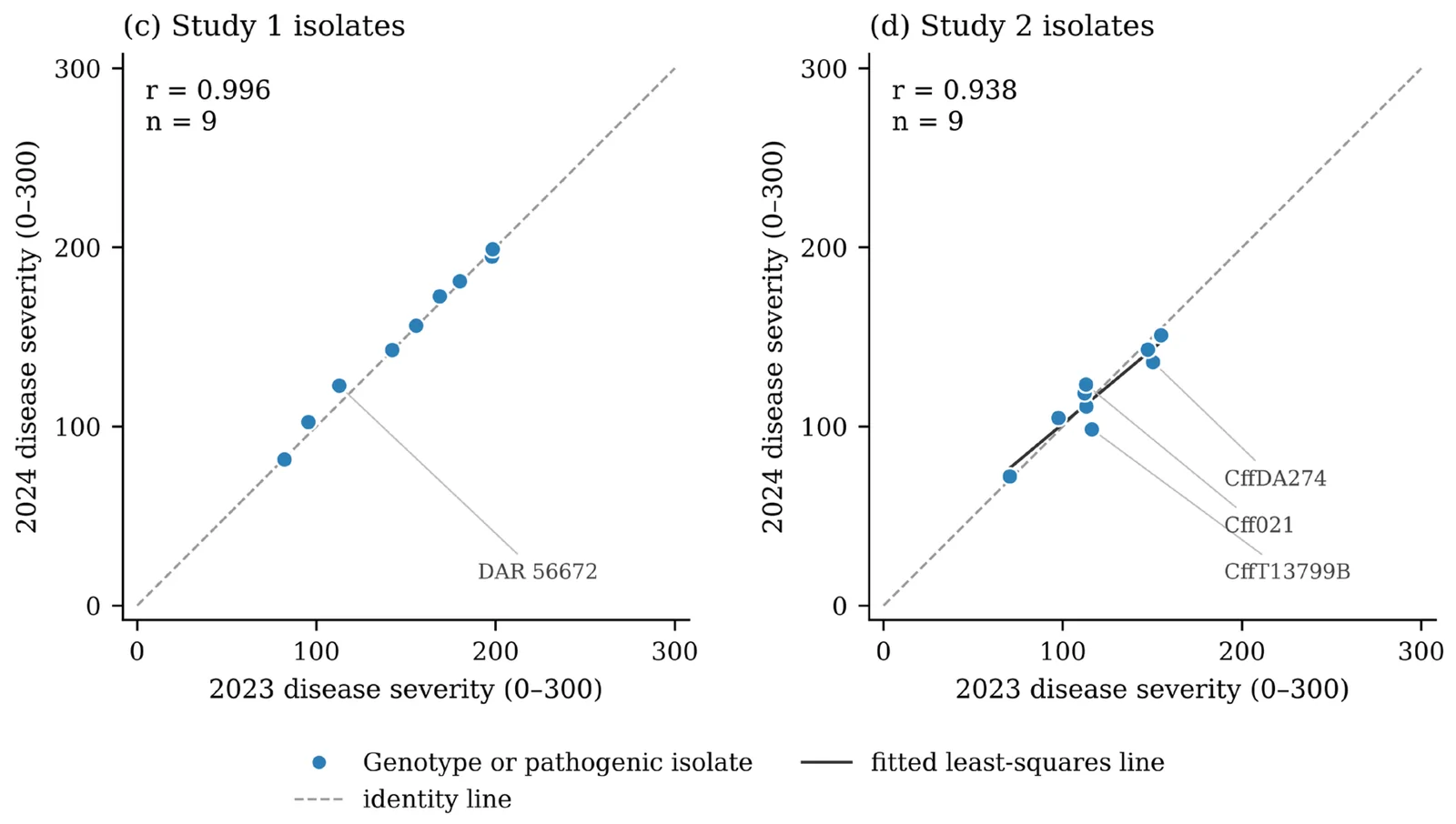
Agreement between the 2023 and 2024 trials for (**a**) the 30 Study 1 genotypes, (**b**) the 12 Study 2 genotypes, (**c**) the nine pathogenic Study 1 isolates and (**d**) the nine pathogenic Study 2 isolates. Each point pairs the trial-specific estimated marginal mean (EMM) for the same genotype or isolate, with 2023 on the x-axis and 2024 on the y-axis. Models were fitted to the nine pathogenic isolates, excluding Cff089. Genotype EMMs are averaged over the observed genotype × isolate combinations. The dashed line is the identity line, and fitted least-squares lines are shown in panels (**b**,**d**). Pearson *r* and *n* are printed in each panel. All panels use the same 0–300 axes.

Genotype EMMs showed between-trial agreement, stronger in Study 1 (Pearson *r* = 0.98, *n* = 30) than in Study 2 (*r* = 0.81, *n* = 12; Figure 3a,b).

Isolate EMMs also showed strong between-trial agreement in both studies (Figure 3c,d). Together with the year tests, these comparisons supported combining the two trials within each study.

### 3.5. Genotype Disease Responses

**Figure 4:**
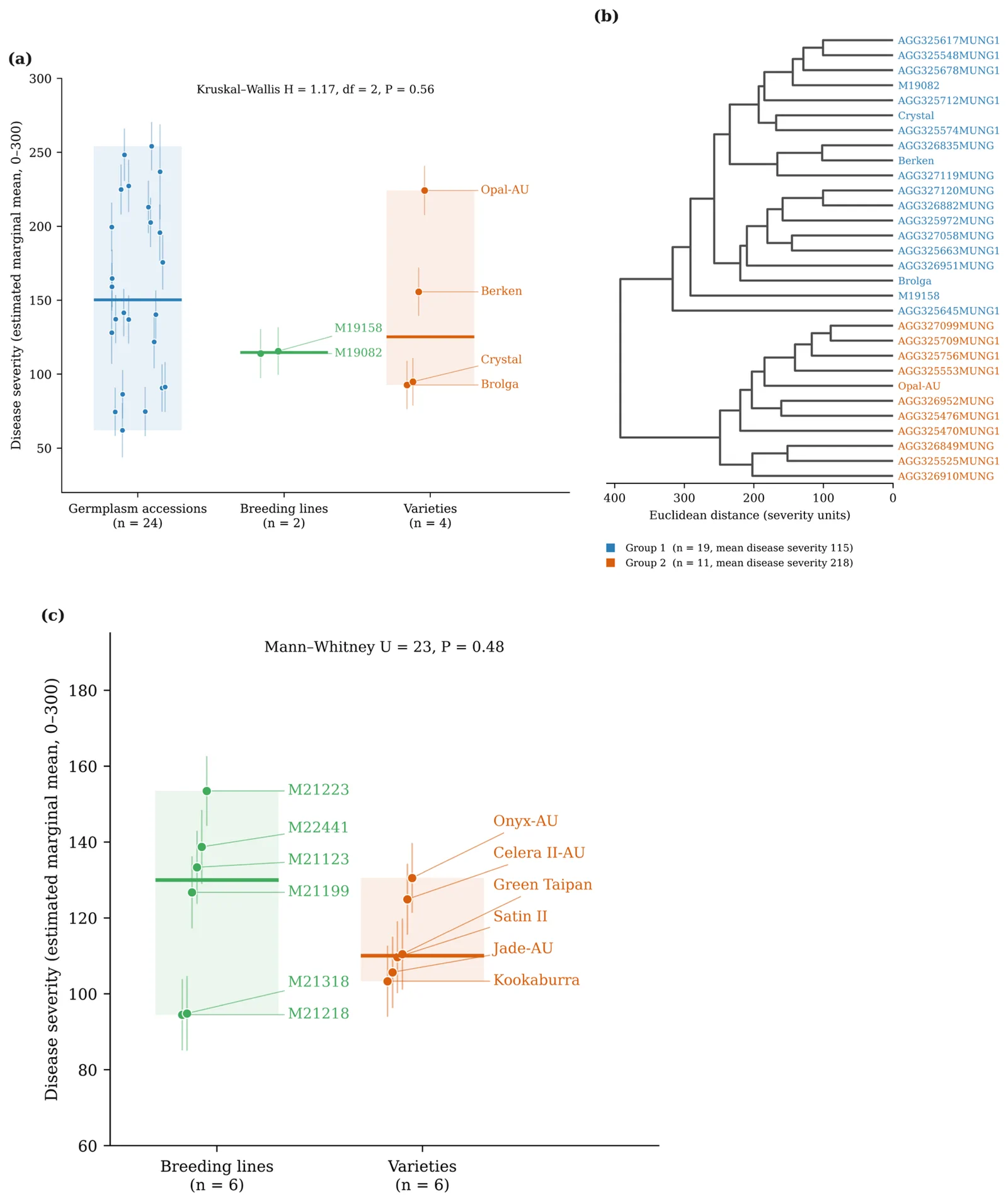
Genotype disease severity by category and descriptive clustering. (**a**) Study 1 included 24 germplasm accessions, two breeding lines and four varieties. Each point is a genotype estimated marginal mean (EMM) disease severity score on the 0–300 scale with its 95% confidence interval. The horizontal bar is the category median and the shaded band shows the category range. The distributions did not differ significantly among categories (Kruskal–Wallis H = 1.17, df = 2, *p* = 0.56). (**b**) Hierarchical clustering of the 30 Study 1 genotype response profiles across nine pathogenic *Curtobacterium flaccumfaciens* pv. *flaccumfaciens* (Cff) isolates, using average linkage and Euclidean distance. Shorter branch distances indicate more similar genotype response profiles. The two descriptive groups contained 19 genotypes with a mean EMM of 115 points and 11 with a mean EMM of 218 points. Fifteen missing genotype × isolate combinations were excluded from pairwise distance calculations and were not imputed. (**c**) Study 2 included six advanced breeding lines and six varieties. Their EMM distributions did not differ significantly (Mann–Whitney U = 23, *p* = 0.48). The clustering is descriptive and no statistical significance is assigned to cluster membership. Varieties are shown by commercial name; analysis identifiers are given in Table 5.

In Study 1, genotype EMMs ranged from 62 points for AGG325972MUNG to 254 points for AGG325525MUNG1 on the 0–300 scale (Table 5). The Study 1 category comparison did not detect a difference in genotype EMM distributions among the 24 germplasm accessions, two breeding lines and four varieties (Kruskal–Wallis H = 1.17, df = 2, *p* = 0.56; Figure 4a), although the breeding-line and variety groups were small.

Descriptive hierarchical clustering identified lower- and higher-disease response groups among the 30 Study 1 genotypes (Figure 4b). These groups describe similarity in response profiles and were not treated as statistically distinct classes.

In Study 2, genotype EMMs ranged from 95 points for the breeding line M21218 to 153 points for M21223 across the nine pathogenic isolates (Table 6). There was no evidence of a difference between the EMM distributions of the six breeding lines and six varieties (Mann–Whitney *U* = 23, *p* = 0.48; Figure 4c). Clustering of the Study 2 genotypes was weak and unstable, so no genotype clusters were interpreted.

**Figure 5:**
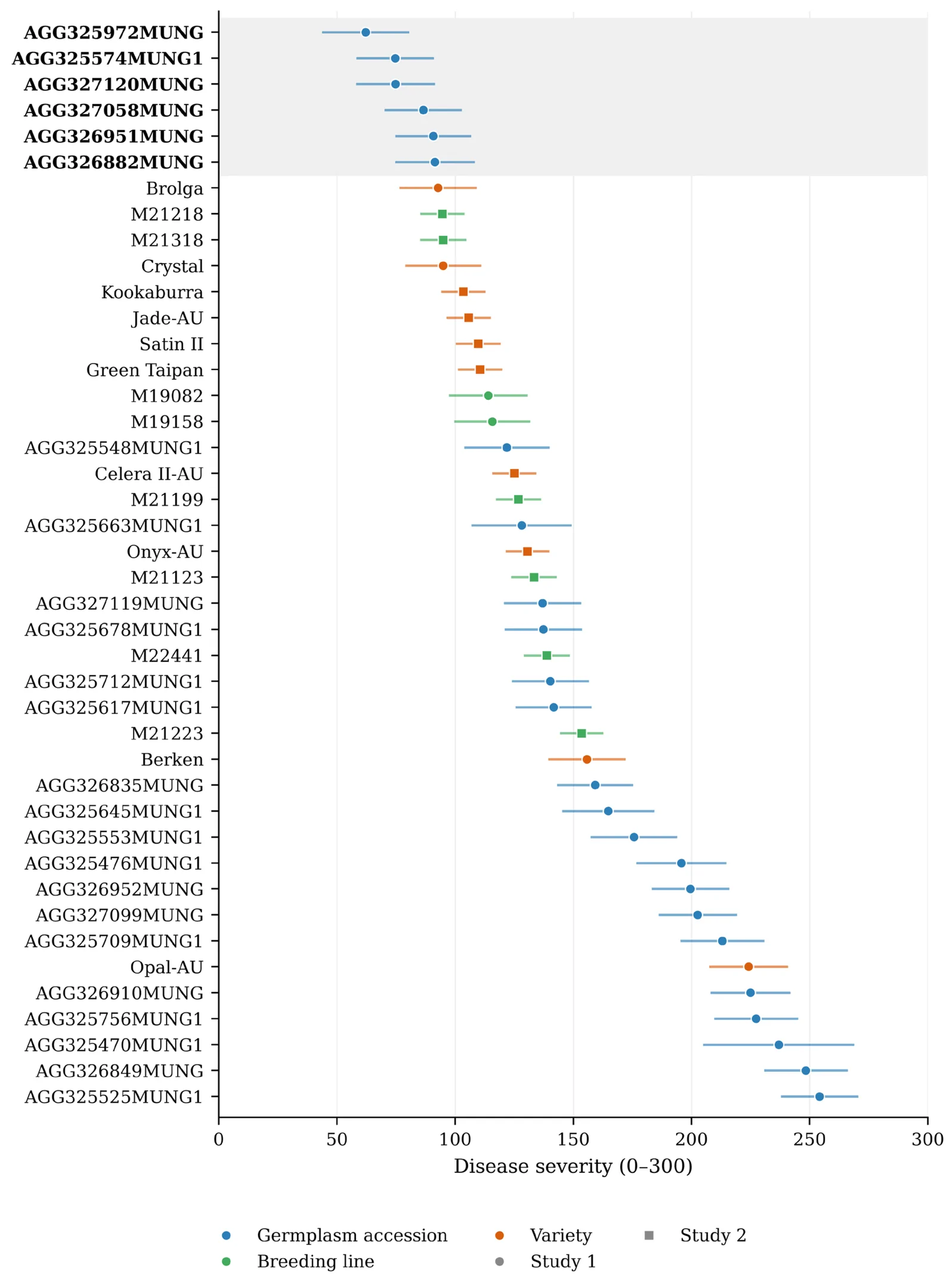
Estimated marginal mean (EMM) disease severity scores for all 42 genotypes, ordered from lowest to highest on the common 0–300 scale. Points are genotype EMMs and horizontal bars are 95% confidence intervals. Colour indicates genotype category and symbol shape indicates study. The shaded region marks the six lowest EMMs, all from Study 1 germplasm accessions. The studies were fitted separately and shared no genotype, so the figure is descriptive and does not test differences between studies. The displayed EMMs are based on the same nine pathogenic *Curtobacterium flaccumfaciens* pv. *flaccumfaciens* (Cff) isolates. Both studies used the same inoculation method and disease-rating scale. Varieties are shown by commercial name.

Figure 5 presents genotype EMMs from the separately analysed studies on a common scale. Within Study 1, the six lowest-disease germplasm accessions did not differ significantly from Brolga, the lowest-EMM variety (Table 5). The display does not support statistical comparisons between studies, which shared no genotypes.

AGG325972MUNG had the lowest genotype EMM in Study 1. Across the eight pathogenic isolates for which observations were available, its observed genotype × isolate cell means ranged from 10 to 115 points.

### 3.6. Isolate-Associated Disease Severity

**Figure 6:**
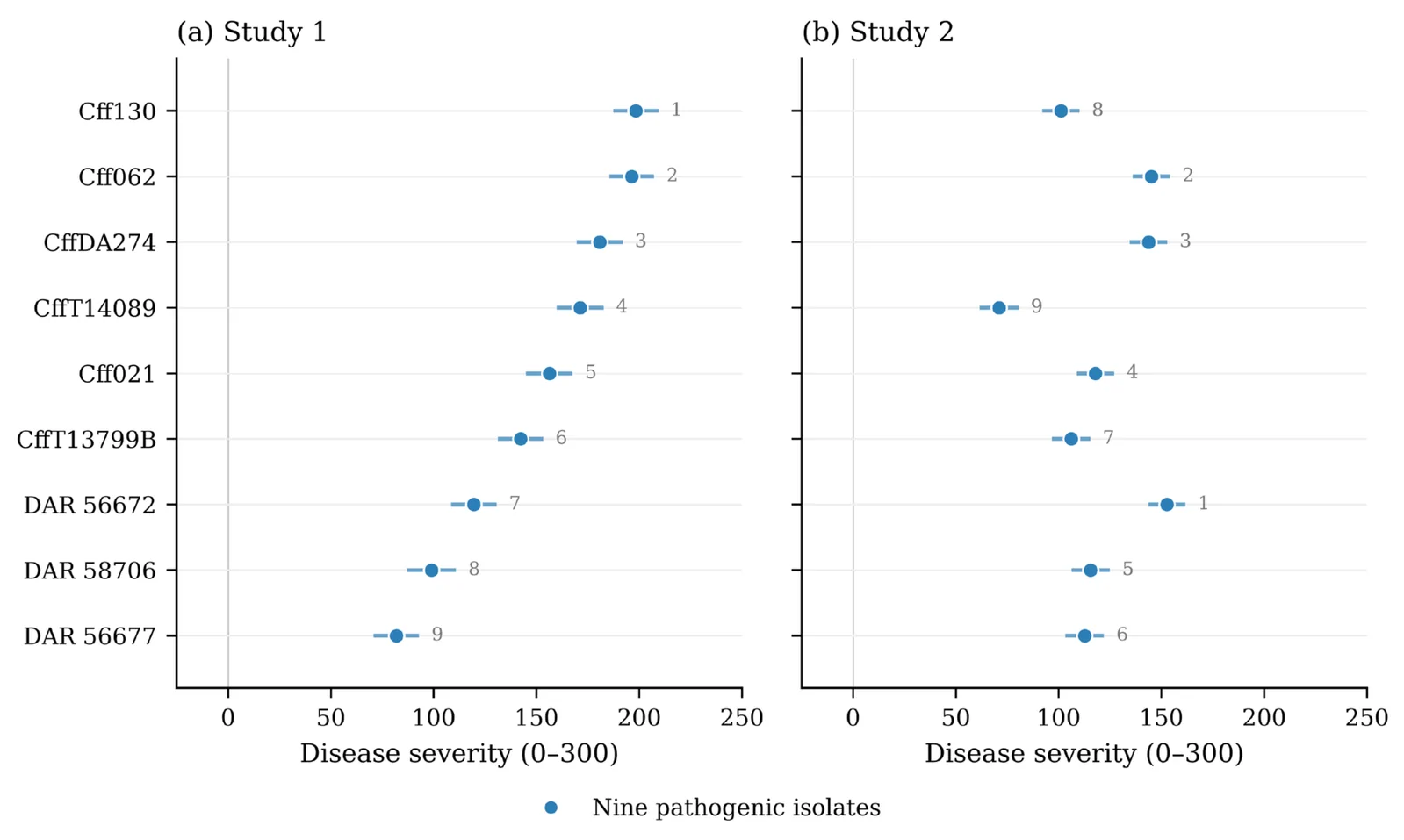
Disease severity associated with the nine pathogenic *Curtobacterium flaccumfaciens* pv. *flaccumfaciens* (Cff) isolates in (**a**) Study 1, across 30 genotypes, and (**b**) Study 2, across 12 genotypes. Each point is the isolate estimated marginal mean (EMM) disease severity score on the 0–300 scale, averaged across observed genotype × isolate combinations and the two trials. Horizontal bars are 95% confidence intervals, and grey numbers give the isolate rank within each study. The studies shared no genotype, so isolate EMMs should not be compared directly between studies. Isolates are ordered by Study 1 disease severity to show changes in rank. The nine pathogenic isolates are shown as blue circles.

The nine pathogenic Cff isolates differed in the disease severity associated with them within each study (Figure 6; Table S7). In Study 1, isolate EMMs ranged from 82 points for DAR 56677 to 198 points for Cff130. Cff130 (198 points) and Cff062 (196 points) were in the highest Tukey group. CffDA274 (181 points) shared a Tukey letter with both of them and with CffT14089 (171 points). In Study 2, isolate EMMs ranged from 71 points for CffT14089 to 153 points for DAR 56672.

Isolates assigned to the same published genetic cluster did not necessarily have similar disease severity. In Study 1, the two cluster 1 isolates, Cff130 and DAR 56672, differed by 79 points. The two cluster 5 isolates, DAR 58706 and CffT13799B, differed by 43 points (Table S7). Collection year was positively associated with disease severity in Study 1 (Spearman *ρ* = 0.83, *p* = 0.006, *n* = 9), where the three isolates collected during the 1986–1987 outbreaks had the lowest disease severity among the pathogenic isolates. No association with collection year was detected in Study 2 (*ρ* = −0.03, *p* = 0.95). The Study 1 association was largely driven by that narrow collection period and does not establish a temporal trend in isolate aggressiveness.

The rank order of the pathogenic isolates changed substantially between the two studies. Only two of the nine pathogenic isolates retained the same rank. The study-specific rankings are shown in Figure 6 and Table S7. Across the nine pathogenic isolates, the study-specific isolate EMMs showed no evidence of a linear association (Pearson *r* = −0.03, 95% CI −0.68 to 0.65, *p* = 0.94) or rank association (Spearman *ρ* = −0.08, *p* = 0.83, *n* = 9). The wide Pearson confidence interval reflects the small number of isolates and limits precision. Including Cff089 in sensitivity models retained significance of genotype, isolate and genotype × isolate interaction in both studies (all *p* < 0.001; Table S5). Pathogenic-isolate rankings were unchanged.

### 3.7. Genotype × Isolate Interaction and Clustering

**Figure 7:**
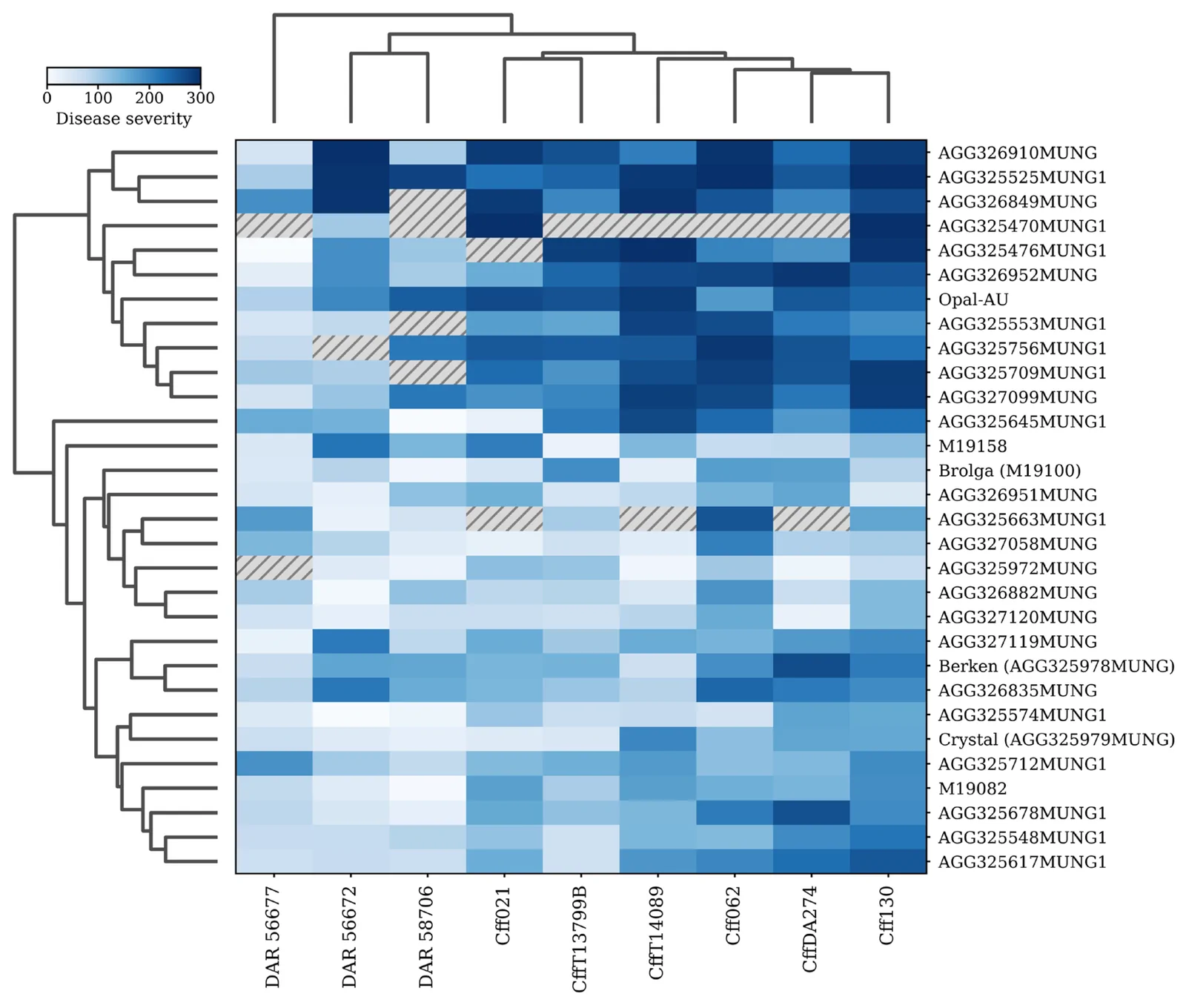
Disease severity of the 30 Study 1 mungbean genotypes against each of the nine pathogenic *Curtobacterium flaccumfaciens* pv. *flaccumfaciens* (Cff) isolates. Each cell is the model-derived estimated marginal mean (EMM) disease severity score for one genotype × isolate combination on the 0–300 scale. Pale cells indicate lower disease and dark cells indicate higher disease. Rows and columns are ordered by descriptive hierarchical clustering using average linkage and Euclidean distance. The 15 hatched grey cells had no complete disease severity observations and were not estimated or imputed. Pairwise distances were calculated only from combinations observed for both members of a pair. Cluster membership is descriptive and has no inferential significance. Brolga (M19100), Crystal (AGG325979MUNG) and Berken (AGG325978MUNG) are shown by commercial name with their analysis identifiers in parentheses. EMM, estimated marginal mean disease severity score.

**Figure 8:**
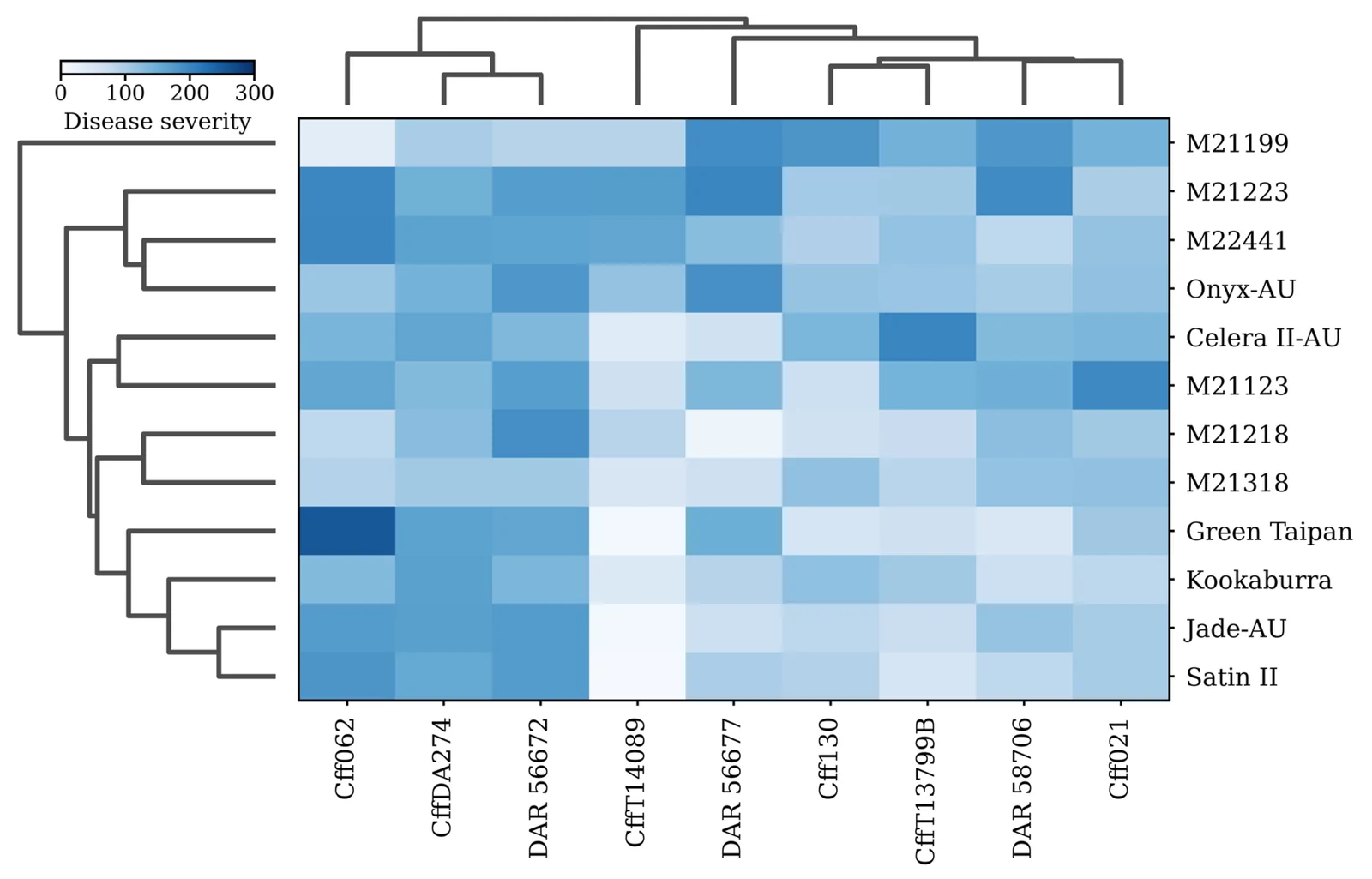
Disease severity of the 12 Study 2 breeding lines and varieties against each of the nine pathogenic *Curtobacterium flaccumfaciens* pv. *flaccumfaciens* (Cff) isolates. Each cell is the model-derived estimated marginal mean (EMM) disease severity score for one genotype × isolate combination on the 0–300 scale. Pale cells indicate lower disease and dark cells indicate higher disease. All 108 combinations were observed. The isolate dendrogram shows descriptive hierarchical clustering of isolate response profiles using average linkage and Euclidean distance. The genotype dendrogram orders rows only. Study 2 genotype clustering was weak, so no genotype groups are interpreted. Neither dendrogram is an inferential test. EMM, estimated marginal mean disease severity score.

Genotype × isolate interaction significantly affected disease severity in both studies (both *p* < 0.001; Table 3 and Table 4). The differences in disease severity among genotypes depended on the isolate used. Some genotype × isolate combinations showed large contrasts. In Study 1, AGG326952MUNG averaged 29 points against DAR 56677 and 294 points against CffDA274. In Study 2, Green Taipan averaged 5 points against CffT14089 and 253 points against Cff062 (Figure 7 and Figure 8). Each value is the mean of four plants, with two plants assessed in each trial. Each contrast was observed in both trials. These replicated contrasts are candidate differential responses, but they do not establish formal pathotypes.

Hierarchical clustering was used to summarise the genotype × isolate response patterns and to order the rows and columns in Figure 7 and Figure 8. Cophenetic correlations for genotype clustering were 0.75 in Study 1 and 0.74 in Study 2. The corresponding values for isolate clustering were 0.85 and 0.80. On complete-profile data, Ward and average linkage gave identical two-group memberships for Study 1 genotypes and Study 2 isolates, but not for Study 1 isolates or Study 2 genotypes. These groupings were therefore treated as descriptive.

Principal component analysis (PCA) provided a second descriptive summary of the response patterns (Figure S1). It included 22 Study 1 genotypes and all 12 Study 2 genotypes with complete profiles across the nine pathogenic isolates. In Study 1, the first component mainly represented overall disease severity, with DAR 56677 showing a different response profile from the other isolates. In Study 2, the first component separated groups of isolates; the second was more closely related to overall genotype disease severity. A missing-data sensitivity analysis retained all 30 Study 1 genotypes and closely reproduced the first component, but agreement was lower for later components. These patterns do not define pathotypes.

Pairwise Spearman correlations showed that isolates did not rank genotypes consistently (Figure S2). Correlations were generally positive in Study 1, whereas 16 of 36 isolate pairs were negatively correlated in Study 2. Similar overall isolate EMMs did not necessarily indicate similar genotype-specific responses. For example, DAR 56677 and DAR 58706 differed by approximately 3 points in Study 2, but their response profiles across the 12 genotypes correlated at only *ρ* = 0.17.

## 4. Discussion

### 4.1. Isolate-Specific Responses and Cross-Study Comparisons

Reliable identification of resistance sources depends on understanding how host genotypes respond to different pathogen isolates. Host genotype, isolate and genotype × isolate interaction significantly affected disease severity in both studies. These interactions show that mungbean responses to Cff isolates varied among genotypes within each study. Screening with a single isolate may therefore give an incomplete assessment of a genotype’s response to other isolates. Diatloff and Imrie [5] reported a significant host × isolate interaction when eight mungbean varieties were tested against two Queensland Cff isolates and recommended screening with more than one pathogenic isolate. The present study extends that work to a larger isolate collection and two independent mungbean genotype sets.

Isolate rankings differed between studies, although isolate responses were strongly correlated between repeated trials within each study. This supports repeatability under the conditions tested but does not explain the between-study differences. Since no genotypes were shared, those differences cannot be attributed specifically to host-panel composition. Therefore, cross-study comparison remains descriptive. Sparks et al. [6] detected disease-severity differences among some pathogenic isolates on Jade-AU. Saad et al. [7] also observed differences in visual symptoms on Opal-AU but noted that using one susceptible cultivar may have limited discrimination among isolates. The present study complements these investigations by demonstrating genotype-specific isolate responses within each study.

### 4.2. Genotype × Isolate Response Patterns

Average disease scores can conceal contrasting responses to individual isolates. AGG326952MUNG had observed mean disease scores of 29 against DAR 56677 and 294 against CffDA274, while Green Taipan averaged 5 against CffT14089 and 253 against Cff062, on the 0–300 scale. These contrasts occurred in both trials. Such genotypes could help develop a differential host set that distinguishes isolates by their disease responses. Valdo et al. [8] identified differential common bean genotypes through their contributions to the genotype × isolate interaction. The mungbean contrasts provide candidates for similar evaluation, rather than evidence of established pathotypes.

Host genetic differences offer a possible explanation for isolate-specific responses. Zia et al. [9] identified resistance-associated markers in common bean that differed among Cff isolates, although the genetic basis of the mungbean responses was not examined here. From the pathogen perspective, genetic cluster membership alone did not predict disease severity: Cff130 and DAR 56672 belonged to the same cluster but differed in their effects within Study 1. This agrees with evidence that phenotypic variation does not necessarily correspond closely to phylogenetic relationships within *Curtobacterium flaccumfaciens* [36]. Isolate selection should therefore consider observed host responses alongside genetic diversity.

Cff089 caused little or no disease, consistent with previous reports of its non-pathogenic behaviour and absence of the pathogenicity-associated plasmid [6,7]. It provided a useful comparison with the nine pathogenic isolates used for the main resistance analyses. Since it was the only plasmid-free isolate tested, its response cannot establish an independent effect of plasmid absence.

### 4.3. Implications for Resistance Screening

AGG325972MUNG had the lowest genotype EMM (62.1) in Study 1. Its observed mean scores ranged from 10 to 115 across the eight pathogenic isolates for which observations were available. Therefore, its low average severity warrants further evaluation against individual isolates. The six lowest-disease accessions did not differ significantly from Brolga, providing a commercial benchmark for disease severity rather than breeding value. Opal-AU had higher mean severity than Brolga and Crystal, consistent with its more susceptible industry rating [10]. However, the screening isolates underlying those ratings were not reported, limiting direct comparison.

The germplasm collection contained both low and high-disease accessions, emphasising the value of evaluating individual responses. Multi-isolate screening has similarly identified wild *Cicer* accessions resistant to several highly pathogenic *Ascochyta rabiei* isolates [37]. The promising mungbean responses require validation before breeding use. Each genotype × isolate combination included four plants across two trials before exclusions, and symptom-free plants were not tested for pathogen presence. Their symptom-free status therefore does not distinguish resistance, asymptomatic infection or unsuccessful infection. Further testing with replicated plants and checks for pathogen infection would help confirm the low-disease responses.

A practical screening panel should represent contrasting host responses as well as pathogen genetic diversity. The combinations identified here provide candidates for evaluating isolates and differential hosts with additional mungbean material and newly collected isolates. Including common low- and high-disease check genotypes, and reporting the isolates used, would make comparisons between screening experiments more informative.

## 5. Conclusions

Tan spot severity was significantly affected by host genotype, Cff isolate and genotype × isolate interaction in both studies. These interactions show that screening with one isolate may give an incomplete assessment of genotype responses to other isolates. The rank order of the nine pathogenic isolates differed between studies, but these differences cannot be attributed specifically to host-panel composition because no genotypes were shared. AGG325972MUNG had the lowest genotype EMM in Study 1, warranting further evaluation against individual pathogenic isolates. Replicated genotype × isolate contrasts also identified candidate differential responses for further testing. These findings support resistance screening with multiple characterised pathogenic Cff isolates representing contrasting host-response profiles, together with further validation of low-disease germplasm as potential sources of tan spot resistance.

## Figures and Tables

**Table 1:** The 42 genotypes evaluated in the two studies. Study 1 comprised 24 germplasm accessions, two breeding lines and four varieties; Study 2 comprised six advanced breeding lines and six varieties. Genotypes are listed by study, then by category, then alphabetically. Category, species and seed source are separate attributes.

Genotype ^a^	Study	Category	Species	Seed Source ^b^
AGG325470MUNG1	Study 1	Germplasm accession	*Vigna radiata*	AGG
AGG325476MUNG1	Study 1	Germplasm accession	*Vigna radiata*	AGG
AGG325525MUNG1	Study 1	Germplasm accession	*Vigna radiata*	AGG
AGG325548MUNG1	Study 1	Germplasm accession	*Vigna radiata*	AGG
AGG325553MUNG1	Study 1	Germplasm accession	*Vigna radiata*	AGG
AGG325574MUNG1	Study 1	Germplasm accession	*Vigna radiata*	AGG
AGG325617MUNG1	Study 1	Germplasm accession	*Vigna radiata*	AGG
AGG325645MUNG1	Study 1	Germplasm accession	*Vigna radiata*	AGG
AGG325663MUNG1	Study 1	Germplasm accession	*Vigna radiata*	AGG
AGG325678MUNG1	Study 1	Germplasm accession	*Vigna radiata*	AGG
AGG325709MUNG1	Study 1	Germplasm accession	*Vigna radiata*	AGG
AGG325712MUNG1	Study 1	Germplasm accession	*Vigna radiata*	AGG
AGG325756MUNG1	Study 1	Germplasm accession	*Vigna radiata*	AGG
AGG325972MUNG	Study 1	Germplasm accession	*Vigna radiata*	AGG
AGG326835MUNG	Study 1	Germplasm accession	*Vigna radiata*	AGG
AGG326849MUNG	Study 1	Germplasm accession	*Vigna radiata*	AGG
AGG326882MUNG	Study 1	Germplasm accession	*Vigna radiata*	AGG
AGG326910MUNG	Study 1	Germplasm accession	*Vigna radiata*	AGG
AGG326951MUNG	Study 1	Germplasm accession	*Vigna radiata*	AGG
AGG326952MUNG	Study 1	Germplasm accession	*Vigna radiata*	AGG
AGG327058MUNG	Study 1	Germplasm accession	*Vigna radiata*	AGG
AGG327099MUNG	Study 1	Germplasm accession	*Vigna radiata*	AGG
AGG327119MUNG	Study 1	Germplasm accession	*Vigna radiata*	AGG
AGG327120MUNG	Study 1	Germplasm accession	*Vigna radiata*	AGG
M19082	Study 1	Breeding line	*Vigna radiata*	QDPI
M19158	Study 1	Breeding line	*Vigna radiata*	QDPI
Berken (AGG325978MUNG)	Study 1	Variety	*Vigna radiata*	AGG
Brolga (M19100)	Study 1	Variety	*Vigna radiata*	QDPI
Crystal (AGG325979MUNG)	Study 1	Variety	*Vigna radiata*	AGG
Opal-AU	Study 1	Variety	*Vigna radiata*	QDPI
M21123	Study 2	Breeding line	*Vigna radiata*	QDPI
M21199	Study 2	Breeding line	*Vigna radiata*	QDPI
M21218	Study 2	Breeding line	*Vigna radiata*	QDPI
M21223	Study 2	Breeding line	*Vigna radiata*	QDPI
M21318	Study 2	Breeding line	*Vigna radiata*	QDPI
M22441	Study 2	Breeding line	*Vigna radiata*	QDPI
Celera II-AU	Study 2	Variety	*Vigna radiata*	QDPI
Green Taipan	Study 2	Variety	*Vigna radiata*	QDPI
Jade-AU	Study 2	Variety	*Vigna radiata*	QDPI
Kookaburra	Study 2	Variety	*Vigna radiata*	QDPI
Onyx-AU	Study 2	Variety	*Vigna mungo*	QDPI
Satin II	Study 2	Variety	*Vigna radiata*	QDPI

Note. ^a^ Analysis identifiers are given in parentheses where they differ from the names used in the text. ^b^ AGG, Australian Grains Genebank (Horsham, Victoria); QDPI, Queensland Department of Primary Industries. The Study 2 genotypes were supplied collectively through National Mungbean Improvement Program (NMIP and QDPI, so no per-genotype source is available for that study.

**Table 2:** The ten *Curtobacterium flaccumfaciens* pv. *flaccumfaciens* isolates evaluated. Isolates are listed in order of collection year. Genetic cluster and plasmid copy number are from previously published metadata.

Isolate	BRIP Accession	Year Collected	Location	Host of Origin	Genetic Cluster ^a^	Plasmid Copies ^b^
DAR 56672	not assigned	1986	Biloela, Qld	*Vigna radiata*	1	2.7
DAR 56677	not assigned	1987	Nemingha, NSW	*Vigna mungo*	3	1.7
DAR 58706	not assigned	1987	Trangie, NSW	*Vigna angularis*	5	1.7
CffT13799B	BRIP 70618	2014	Warwick, Qld	*Vigna radiata*	5	not tested
CffT14089	BRIP 70621	2015	Biloela, Qld	*Vigna radiata*	4	2.5
Cff021	BRIP 70604	2018	Mullaley, NSW	*Vigna radiata*	6	1.7
Cff062	BRIP 70610	2018	Mullaley, NSW	*Vigna radiata*	2	2.0
Cff089	not assigned	2018	Brookstead, Qld	*Vigna radiata*	admixed (2 and 5)	0.0 (no plasmid detected)
Cff130	BRIP 70614	2018	Wooroolin, Qld	*Vigna radiata*	1	2.4
CffDA274	BRIP 70623	2019	Brookstead, Qld	*Vigna radiata*	2	2.4

Note. ^a^ Cluster in the six-cluster population structure of Sparks et al. [6]. Cff089 is of admixed ancestry between clusters 2 and 5. ^b^ Plasmid pCff119 copy numbers estimated by droplet digital PCR (ddPCR) [7]. Values are ddPCR-derived estimates of the plasmid pCff119-to-chromosome copy-number ratio. No plasmid was detected in Cff089. CffT13799B was not tested. Isolates with a DAR designation were obtained from the DAR Culture Collection, Orange, New South Wales; the remainder were sourced from a collection at the Centre for Crop Health, University of Southern Queensland. Qld, Queensland; NSW, New South Wales; BRIP, Queensland Plant Pathology Herbarium.

**Table 3:** Tests of fixed effects for the disease severity score in Study 1 (30 mungbean genotypes inoculated with each of the nine pathogenic *Curtobacterium flaccumfaciens* pv. *flaccumfaciens* isolates). The disease severity score was the sum of the three trifoliate ratings on a 0–300 scale. It was analysed with a linear mixed model containing genotype, isolate, year and genotype × isolate interaction as fixed effects and pot as a random intercept. The model was fitted by restricted maximum likelihood.

Effect	*ndf*	*ddf*	*F*	*p*
Genotype ^a^	29	685	44.00	<0.001
Isolate ^a^	8	233	54.74	<0.001
Year ^a^	1	245	0.40	0.529
Genotype × Isolate ^b^	217	553	4.84	<0.001

Note. ^a^ Genotype, isolate and year were tested by joint tests of estimated marginal means (EMMs). For each factor, the null hypothesis was that all EMMs of that factor were equal. ^b^ In Study 1, 15 of 270 genotype × isolate combinations had no complete disease severity score, so the genotype × isolate interaction was tested over 217 estimable degrees of freedom. Conventional Type III tests are shown in Table S2 for comparison. Kenward–Roger inference was used throughout. ndf, numerator degrees of freedom; ddf, approximate denominator degrees of freedom. *p* values below 0.001 are shown as <0.001.

**Table 4:** Tests of fixed effects for the disease severity score in Study 2 (12 breeding lines and varieties inoculated with each of the nine pathogenic *Curtobacterium flaccumfaciens* pv. *flaccumfaciens* isolates). The disease severity score was the sum of the three trifoliate ratings on a 0–300 scale. It was analysed with a linear mixed model containing genotype, isolate, year and genotype × isolate interaction as fixed effects and pot as a random intercept. The model was fitted by restricted maximum likelihood.

Effect	*ndf*	*ddf*	*F*	*p*
Genotype ^a^	11	263	14.76	<0.001
Isolate ^a^	8	99	30.14	<0.001
Year ^a^	1	105	0.55	0.462
Genotype × Isolate	88	262	9.15	<0.001

Note. ^a^ Genotype, isolate and year were tested by joint tests of estimated marginal means (EMMs). For each factor, the null hypothesis was that all EMMs of that factor were equal. All 108 genotype × isolate combinations were observed in Study 2, and conventional Type III tests gave closely similar results (Table S2). Kenward–Roger inference was used throughout. ndf, numerator degrees of freedom; ddf, approximate denominator degrees of freedom. *p*-values below 0.001 are shown as <0.001.

**Table 5:** Disease severity of the 30 Study 1 mungbean genotypes, listed from lowest to highest estimated marginal mean (EMM). The EMM is the model-derived mean disease severity score of a genotype on the 0–300 scale, averaged across the nine pathogenic isolates using the observed genotype × isolate combinations and the two trials. Genotype category and seed source are separate attributes.

Genotype ^a^	Category	Seed Source ^b^	EMM	SE	95% CI	Tukey ^c^	Rank	Descriptive Group ^d^
AGG325972MUNG	Germplasm accession	AGG	62.1	9.4	43.7 to 80.5	a	1	Group 1, lower disease
AGG325574MUNG1	Germplasm accession	AGG	74.6	8.3	58.2 to 90.9	ab	2	Group 1, lower disease
AGG327120MUNG	Germplasm accession	AGG	74.8	8.5	58.1 to 91.4	ab	3	Group 1, lower disease
AGG327058MUNG	Germplasm accession	AGG	86.5	8.3	70.1 to 102.8	abc	4	Group 1, lower disease
AGG326951MUNG	Germplasm accession	AGG	90.7	8.2	74.7 to 106.8	abc	5	Group 1, lower disease
AGG326882MUNG	Germplasm accession	AGG	91.4	8.6	74.5 to 108.3	abc	6	Group 1, lower disease
Brolga (M19100)	Variety	QDPI	92.7	8.3	76.4 to 109.1	abc	7	Group 1, lower disease
Crystal (AGG325979MUNG)	Variety	AGG	94.9	8.2	78.8 to 111.0	abcd	8	Group 1, lower disease
M19082	Breeding line	QDPI	114.0	8.5	97.3 to 130.6	bcde	9	Group 1, lower disease
M19158	Breeding line	QDPI	115.7	8.2	99.6 to 131.7	bcdef	10	Group 1, lower disease
AGG325548MUNG1	Germplasm accession	AGG	121.8	9.2	103.8 to 139.9	cdefg	11	Group 1, lower disease
AGG325663MUNG1	Germplasm accession	AGG	128.1	10.8	106.9 to 149.3	cdefgh	12	Group 1, lower disease
AGG327119MUNG	Germplasm accession	AGG	137.0	8.3	120.6 to 153.3	defgh	13	Group 1, lower disease
AGG325678MUNG1	Germplasm accession	AGG	137.3	8.3	120.9 to 153.6	defgh	14	Group 1, lower disease
AGG325712MUNG1	Germplasm accession	AGG	140.3	8.3	123.9 to 156.6	efgh	15	Group 1, lower disease
AGG325617MUNG1	Germplasm accession	AGG	141.6	8.2	125.5 to 157.7	efgh	16	Group 1, lower disease
Berken (AGG325978MUNG)	Variety	AGG	155.7	8.3	139.3 to 172.1	efghi	17	Group 1, lower disease
AGG326835MUNG	Germplasm accession	AGG	159.2	8.2	143.1 to 175.2	fghij	18	Group 1, lower disease
AGG325645MUNG1	Germplasm accession	AGG	164.7	9.9	145.3 to 184.2	ghijk	19	Group 1, lower disease
AGG325553MUNG1	Germplasm accession	AGG	175.6	9.4	157.3 to 194.0	hijkl	20	Group 2, higher disease
AGG325476MUNG1	Germplasm accession	AGG	195.7	9.7	176.7 to 214.7	ijklm	21	Group 2, higher disease
AGG326952MUNG	Germplasm accession	AGG	199.6	8.3	183.2 to 215.9	ijklm	22	Group 2, higher disease
AGG327099MUNG	Germplasm accession	AGG	202.6	8.5	186.0 to 219.2	jklmn	23	Group 2, higher disease
AGG325709MUNG1	Germplasm accession	AGG	213.0	9.0	195.3 to 230.8	klmno	24	Group 2, higher disease
Opal-AU (Opal)	Variety	QDPI	224.2	8.5	207.5 to 240.8	mno	25	Group 2, higher disease
AGG326910MUNG	Germplasm accession	AGG	224.9	8.6	208.0 to 241.8	mno	26	Group 2, higher disease
AGG325756MUNG1	Germplasm accession	AGG	227.3	9.0	209.6 to 245.0	mno	27	Group 2, higher disease
AGG325470MUNG1	Germplasm accession	AGG	236.9	16.3	204.9 to 268.9	lmno	28	Group 2, higher disease
AGG326849MUNG	Germplasm accession	AGG	248.4	9.0	230.7 to 266.1	no	29	Group 2, higher disease
AGG325525MUNG1	Germplasm accession	AGG	254.2	8.3	237.8 to 270.5	o	30	Group 2, higher disease

Note. ^a^ Analysis identifiers differ from the names used in the text for four varieties: Brolga is M19100, Crystal is AGG325979MUNG, Berken is AGG325978MUNG and Opal-AU is Opal. ^b^ AGG, Australian Grains Genebank; QDPI, Queensland Department of Primary Industries. ^c^ Genotypes sharing a letter did not differ at *α* = 0.05 in Tukey-adjusted pairwise comparisons. ^d^ The two cluster groups are the descriptive groups shown in Figure 4b and have no inferential significance. EMM, estimated marginal mean disease severity score; SE, standard error; 95% CI, 95% confidence interval; Rank, position within Study 1 from lowest to highest disease severity. Study 1 included 30 genotypes and nine pathogenic isolates in this analysis.

**Table 6:** Disease severity of the 12 Study 2 breeding lines and varieties, listed from lowest to highest estimated marginal mean (EMM). The EMM is the model-derived mean disease severity score of a genotype on the 0–300 scale, averaged across the nine pathogenic isolates and the two trials. Study 2 comprised six breeding lines and six varieties. Study 1 and Study 2 shared no genotype and were analysed separately, so EMMs should be compared only within a study.

Genotype	Species	Category	EMM	SE	95% CI	Tukey ^a^	Rank
M21218	*Vigna radiata*	Breeding line	94.5	4.8	85.2 to 103.9	a	1
M21318	*Vigna radiata*	Breeding line	94.9	5.0	85.0 to 104.7	a	2
Kookaburra	*Vigna radiata*	Variety	103.4	4.8	94.0 to 112.7	a	3
Jade-AU	*Vigna radiata*	Variety	105.7	4.8	96.3 to 115.1	ab	4
Satin II	*Vigna radiata*	Variety	109.7	4.8	100.2 to 119.2	abc	5
Green Taipan	*Vigna radiata*	Variety	110.5	4.8	101.1 to 119.9	abc	6
Celera II-AU	*Vigna radiata*	Variety	124.9	4.8	115.6 to 134.3	bcd	7
M21199	*Vigna radiata*	Breeding line	126.8	4.8	117.2 to 136.3	bcd	8
Onyx-AU	*Vigna mungo*	Variety	130.6	4.7	121.4 to 139.8	cd	9
M21123	*Vigna radiata*	Breeding line	133.3	4.9	123.7 to 143.0	de	10
M22441	*Vigna radiata*	Breeding line	138.7	5.0	129.0 to 148.5	de	11
M21223	*Vigna radiata*	Breeding line	153.5	4.7	144.3 to 162.7	e	12

Note. ^a^ Genotypes sharing a letter did not differ at *α* = 0.05 in Tukey-adjusted pairwise comparisons. Letters are not comparable with Table 5. Onyx-AU is a black gram (*Vigna mungo*) variety; every other Study 2 genotype is *Vigna radiata*. EMM, estimated marginal mean disease severity score; SE, standard error; 95% CI, 95% confidence interval; Rank, position within Study 2 from lowest to highest disease severity. Study 2 included 12 genotypes and nine pathogenic isolates in this analysis.

## Data Availability

The plant-level data supporting the findings of this study are provided in Supplementary Data S1. Mungbean genotype accessions are maintained by the Australian Grains Genebank (Horsham, Victoria) and the Queensland Department of Primary Industries and may be obtained from those institutions subject to their respective material transfer policies.

## Supplementary Materials

The following supporting information can be downloaded at: https://www.mdpi.com/article/10.3390/plants15203148/s1.
