# Supplementary material for: Genotype × Isolate Interactions for Tan Spot Resistance Screening in Mungbean
Source: Plants (Basel). 2026 Oct 15;15(20):3148. doi: 10.3390/plants15203148 (PMC13708807; doi:10.3390/plants15203148)
Supplement: Supplementary file 1 [file plants-15-03148-s001.zip › plants-4555164-Supplementary.pdf]

Supplementary Information

for

Genotype × Isolate Interactions for Tan Spot Resistance Screening in Mungbean

Krishna Chaitanya Alamuru[1]*, Noel L. Knight[1], Prue Bottomley[1,2], Merrill Ryan[3], Levente Kiss[1], Anke Martin[1]*

[1]Centre for Crop Health, School of Science, Engineering and Digital Technologies, University of Southern Queensland, Toowoomba, QLD 4350, Australia.

[2]Metagen Pty Ltd, Gatton, QLD, 4343, Australia.

[3]Queensland Department of Primary Industries, Warwick, QLD 4370, Australia.

Corresponding Authors

* Krishna Chaitanya Alamuru – krishnachaitanya.alamuru@unisq.edu.au

* Anke Martin – anke.martin@unisq.edu.au

Contents: Supplementary Methods S1–S7, Tables S1–S7 and Figures S1–S2; Supplementary Data S1 (separate Excel file). Supplementary Data S1 provides the plant-level disease-severity data for both studies in one Excel sheet. Tri1, Tri2 and Tri3 are the first three trifoliate scores (0–100 each); Severity_sum is their sum (0–300). Blank scores denote unavailable observations; Table S1 distinguishes pre-inoculation establishment losses from incomplete leaf assessments. Analysis_included identifies complete records eligible for analysis; the primary nine-isolate analyses additionally exclude Cff089. File: *plants-4555164_Data_S1.xlsx.*

Supplementary Methods

Reference numbers correspond to the reference list in the main manuscript.

S1. Software versions

Mixed-model analyses used R v4.5.2 [15], lme4 v1.1-38 [16], lmerTest v3.2-1 [17], emmeans v2.0.4 [18] and pbkrtest v0.5.5 [19]. Compact-letter groupings used multcompView v0.1-11. Clustering, PCA and figures used Python v3.12.14, NumPy v2.3.5, pandas v2.2.3, SciPy v1.18.1 [20], scikit-learn v1.9.1 [21] and Matplotlib v3.11.2 [22].

S2. Fixed-effect tests and missing genotype × isolate cells

Study 1 lacked complete plant scores for 15 of 270 genotype × isolate combinations. Genotype, isolate and year main effects were tested using joint tests of estimable marginal means with emmeans weights = "flat", which gives equal weight to observed cells and zero weight to unobserved cells. This avoided the coding-dependent Type III main-effect hypotheses arising from missing cells.

The genotype × isolate interaction was tested separately using the default joint-test reference grid over its full estimable space: 217 interaction degrees of freedom in Study 1 and 88 in Study 2. Thus, the main-effect and interaction tests did not use the same weighting procedure. All 108 combinations were observed in Study 2. The comparison with conventional Type III Kenward–Roger tests is presented in Table S2.

S3. Year interactions and Study 2 sensitivity models

Nested-model comparisons assessed genotype × year, isolate × year, these two terms jointly, and genotype × isolate × year using Kenward–Roger approximate F tests (Table S3).

For Study 2, the primary model was refitted with genotype × year added as a fixed effect. A separate all-random model contained genotype, isolate, genotype × isolate, year, genotype × year, isolate × year and pot to estimate interaction variance. These sensitivity models were distinct from both the primary inferential model and the standard variance-partitioning model.

S4. Variance components

The standard variance-partitioning model contained random intercepts for genotype, isolate, genotype × isolate, year and pot. Total variance was the sum of these components and residual variance. Component percentages were calculated from variance estimates, not fixed-effect F statistics (Table S4). The all-ten-isolate comparison included Cff089 using the same structure.

S5. Clustering, PCA and response-profile comparisons

For Study 1, each pairwise distance used only coordinates observed for both members of the pair and was rescaled for the number of shared observations. This procedure was applied to both genotype and isolate clustering; no unobserved cell was imputed. Solutions with two to six groups were evaluated using silhouette coefficients [30]. Ward linkage [31] on the complete-case submatrix was a sensitivity analysis [32]. Cophenetic correlations assessed preservation of pairwise distances by each dendrogram [33].

Primary PCA used complete genotype profiles across the nine pathogenic isolates. The missing-data sensitivity analysis used nonlinear iterative partial least squares (NIPALS) to retain all 30 Study 1 genotypes, skipping unobserved entries rather than replacing the recorded data.

Spearman correlations between pathogenic isolate response profiles used genotype × isolate cell EMMs. Study 1 used pairwise-complete genotypes (n = 24–29); Study 2 used all 12 genotypes. Cff089 was excluded because its near-zero response profile was uninformative for genotype ranking.

Study 1 category comparisons included germplasm accessions, breeding lines and varieties; Study 2 comparisons included breeding lines and varieties. These comparisons used genotype-level estimates, not individual plants as independent category observations.

S6. Trial agreement and influence checks

Within each study and trial, genotype and isolate EMMs were obtained from the primary fixed-effect structure without year, retaining the pot random intercept and observed-cell weighting. Pearson correlations measured linear association; Spearman correlations compared ranks. Pearson 95% confidence intervals used the Fisher z transformation.

Because correlation does not measure equality of severity between trials, the mean and range of trial-to-trial EMM differences were also examined. Correlations were recalculated after omitting each genotype or isolate in turn to assess influence; these checks did not remove levels from the reported full analysis. These comparisons used the nine pathogenic isolates.

S7. Cff089 and incomplete leaf-score sensitivity analyses

Sensitivity models included all ten isolates, adding Cff089 to the primary nine-pathogenic-isolate panel, with the same fixed and random effects and inference (Table S5). Variance components were similarly compared using the same all-random model structure (Table S4).

For the 17 partially scored inoculated plants, each missing trifoliate score was assigned either 0 or 100 in separate sensitivity datasets. Recorded leaf scores were unchanged; the primary model was refitted for each study using only the nine pathogenic isolates. The 126 records associated with failed germination or removal of late-germinating seedlings before inoculation remained excluded. Genotype and isolate EMM ranks were compared with the primary analysis using Spearman correlations (Table S6). Assigned scores did not replace the recorded observations in Supplementary Data S1.

Cross-study isolate correlations used only the nine pathogenic isolates because including Cff089, with near-zero EMMs in both studies, could artificially increase the correlation. These correlations described the observed cross-study relationship; they did not identify host-panel composition as its cause.

Table S1. Accounting of plants sown, pre-inoculation establishment losses and incomplete trifoliate assessments.

Each plant is assigned to one outcome, so the three outcome columns sum to the number of plants sown in that row. Disease severity was analysed only for inoculated plants with a complete score on the 0–300 scale. Complete-score totals include Cff089; the primary nine-pathogenic-isolate analysis used 957 plants in Study 1 and 412 in Study 2.

**Study**	**Trial**	Plants sown	**Complete scores**[a]	**Unavailable before inoculation**[b]	**Excluded with only one or two trifoliate scores available**
Study 1	2023	600	513	83	4
Study 1	2024	600	564	36	0
Study 1	Both trials	1200	1077	119	4
Study 2	2023	240	220	7	13
Study 2	2024	240	240	0	0
Study 2	Both trials	480	460	7	13
Both studies	Both trials	1680	1537	126	17

[a] The complete-score totals include 48 Study 2 plants inoculated with Cff089, 24 in each trial. These were established, inoculated and assessed plants that showed no disease. The Study 2 score sheets left these observations blank rather than entering zero, and they were entered as zero severity because they represented a documented absence of disease rather than a missing assessment. The equivalent Study 1 scores had been entered directly at assessment: 118 of the 120 Study 1 Cff089 plants scored zero and two carried non-zero recorded scores. Their cause could not be determined, and both were retained as observed.

Among plants inoculated with pathogenic isolates, 33 scored zero across 19 genotype × isolate combinations. No combination had two zero-scored plants within the same trial. The paired-block plant showed symptoms in 29 cases and lacked a complete score in four. Zero scores occurred in both trials for 14 combinations. Symptoms in paired plants confirm that the treatment could produce disease, but do not establish infection in each symptom-free plant.

[b] These plants were unavailable before inoculation because seeds failed to germinate or late-germinating seedlings were removed. Separate counts for these two causes were not recorded. No Cff089 record falls in this group. Among partially scored plants, missing trifoliates were generally observed to reflect plant development or leaf loss rather than disease. Leaves dried in response to inoculation were scored as diseased.

Table S2. Comparison of conventional Type III tests with the joint tests of estimated marginal means reported in Tables 3 and 4 for the nine pathogenic isolates.

The disease severity score, the summed severity of the three trifoliate leaves of each plant on a 0–300 scale, was analysed with a linear mixed model containing genotype, isolate, year and the genotype × isolate interaction as fixed effects and pot as a random intercept, fitted by restricted maximum likelihood.

Study	Effect	*ndf*	*ddf*	*F*	*P*
Study 1	Genotype	29	683	43.18	<0.001
Study 1	Isolate	8	248	53.59	<0.001
Study 1	Year	1	245	0.15	0.698
Study 1	Genotype × Isolate	217	575	4.84	<0.001
Study 2	Genotype	11	260	14.29	<0.001
Study 2	Isolate	8	106	31.40	<0.001
Study 2	Year	1	108	0.05	0.821
Study 2	Genotype × Isolate	88	247	9.11	<0.001

In Study 1, 15 genotype × isolate combinations were not observed. The Type III main-effect hypotheses then depend on which aliased interaction coefficients are dropped from the model and are not uniquely defined, which is why the joint tests of estimated marginal means given in Table 3 are reported in preference. Study 2 has no unobserved combinations and the two approaches agree closely there.

Kenward–Roger inference was used throughout to account for the small-sample and unbalanced mixed-model design. *ndf*, numerator degrees of freedom; *ddf*, Kenward–Roger approximate denominator degrees of freedom. *P* values below 0.001 are shown as <0.001.

Table S3. Year main effect and year-interaction tests for the nine pathogenic isolates in both studies.

Interactions involving year were tested by Kenward–Roger approximate *F* tests comparing nested models. These tests assessed between-trial effects and interactions relevant to combining the two trials.

Study	Term	*ndf*	*ddf*	*F*	*P*
Study 1	Genotype × Isolate × Year	215	317	0.11	>0.999
Study 1	Genotype × Year and Isolate × Year jointly	37	629	0.20	>0.999
Study 1	Genotype × Year	29	608	0.23	>0.999
Study 1	Isolate × Year	8	240	0.07	>0.999
Study 1	Year[a]	1	245	0.40	0.529
Study 2	Genotype × Isolate × Year	83	152	0.68	0.973
Study 2	Genotype × Year and Isolate × Year jointly	19	198	1.63	0.052
Study 2	Genotype × Year	11	251	1.88	0.042
Study 2	Isolate × Year	8	100	1.32	0.243
Study 2	Year[a]	1	105	0.55	0.462

[a] The year main effect is reported by the same joint test of estimated marginal means used in Tables 3 and 4, so that one effect carries one value throughout.

Kenward–Roger inference was used throughout to account for the small-sample and unbalanced mixed-model design. *ndf*, numerator degrees of freedom; *ddf*, Kenward–Roger approximate denominator degrees of freedom. *P* values below 0.001 are shown as <0.001.

Table S4. Variance components for disease severity in both studies, for the primary nine-pathogenic-isolate panel and the all-ten-isolate comparison.

A model fitting genotype, isolate, the genotype × isolate interaction, year and pot as random effects was used to partition the variance in disease severity. The genotype variance component represents consistent differences among genotypes across isolates, whereas the genotype × isolate component represents isolate-specific deviations from those average responses. Year is fitted as a random effect in this variance-partitioning model only; in the primary model of each study it remains a fixed effect. The same model was fitted with the nine pathogenic isolates and with all ten isolates including Cff089. Percentages use the sum of all variance components, including residual variance. Year variance was estimated at zero in all four fits, producing boundary (singular) fits. Values are rounded; percentages may not sum to exactly 100%.

Study	Source	Variance nine pathogenic	% nine pathogenic	Variance all 10	% all 10
Study 1	Isolate	1616	17.3	3653	34.6
Study 1	Genotype × Isolate	2356	25.3	2388	22.6
Study 1	Genotype	2900	31.1	2284	21.7
Study 1	Residual	2133	22.9	1935	18.3
Study 1	Pot	317	3.4	289	2.7
Study 1	Year	0	0.0	0	0.0
Study 2	Isolate	529	17.1	1863	44.7
Study 2	Genotype × Isolate	1642	53.1	1492	35.8
Study 2	Residual	678	21.9	605	14.5
Study 2	Genotype	137	4.4	110	2.6
Study 2	Pot	108	3.5	97	2.3
Study 2	Year	0	0.0	0	0.0

Because the fixed-effect F statistics in Tables 3 and 4 test different hypotheses on different numerator degrees of freedom, these variance components, and not the F statistics, are the basis for statements about the relative contribution of each source.

Table S5. Sensitivity analysis including all ten isolates.

Both models were refitted with Cff089 included, using the same fixed and random structure, equally weighted EMMs for the main-effect joint tests, and all estimable degrees of freedom for the interaction test, as in the primary analysis. This comparison assesses the influence of including the non-pathogenic control alongside the nine pathogenic isolates analysed in Tables 3 and 4.

**Study**	**Effect**	***ndf***	***ddf***	***F***	***P***
Study 1	Genotype	29	774	43.37	<0.001
Study 1	Isolate	9	247	136.71	<0.001
Study 1	Year	1	273	0.23	0.629
Study 1	Genotype × Isolate	246	623	5.30	<0.001
Study 2	Genotype	11	295	14.70	<0.001
Study 2	Isolate	9	111	106.19	<0.001
Study 2	Year	1	116	0.31	0.581
Study 2	Genotype × Isolate	99	294	9.30	<0.001

In Study 1, 15 of the 300 genotype × isolate combinations were not observed, so the interaction was tested over its estimable space of 246 degrees of freedom.

Kenward–Roger inference was used throughout to account for the small-sample and unbalanced mixed-model design. *ndf*, numerator degrees of freedom; *ddf*, Kenward–Roger approximate denominator degrees of freedom. *P* values below 0.001 are shown as <0.001.

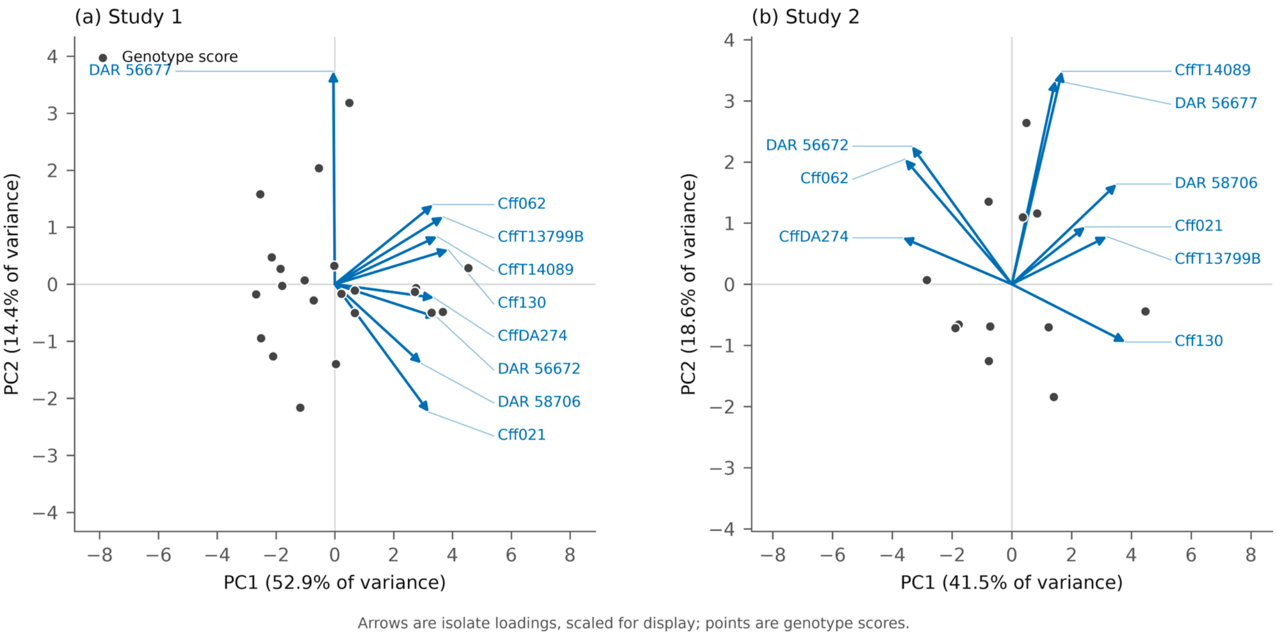

Figure S1. Principal component analysis of the genotype × isolate disease-severity matrices for (a) Study 1 and (b) Study 2. The analysis included the nine pathogenic *Curtobacterium flaccumfaciens* pv. *flaccumfaciens* (Cff) isolates and only genotypes with complete profiles across those isolates: 22 of 30 genotypes in Study 1 and all 12 genotypes in Study 2. Each isolate variable was centred and scaled to unit variance before analysis. Black points show genotype scores and blue arrows show isolate loadings, scaled for display. PC1 and PC2 explained 52.9% and 14.4% of the variance in Study 1 and 41.5% and 18.6% in Study 2, respectively. The analysis is descriptive. PC1, first principal component; PC2, second principal component.

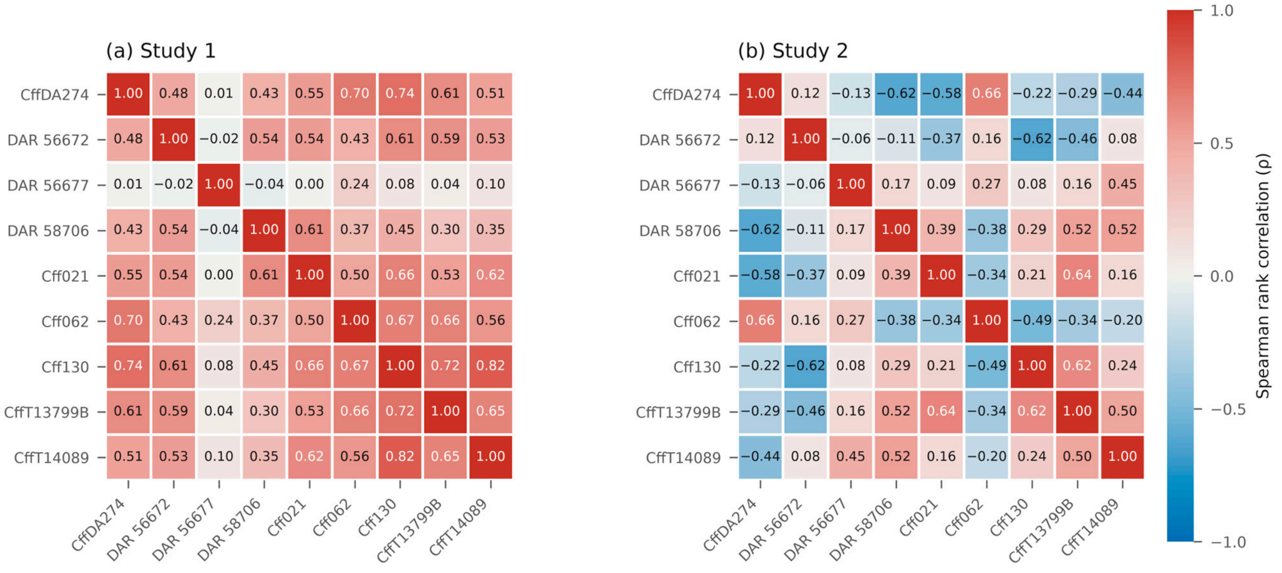

Figure S2. Spearman rank correlations among disease-severity response profiles of the nine pathogenic *Curtobacterium flaccumfaciens* pv. *flaccumfaciens* (Cff) isolates across genotypes in (a) Study 1 and (b) Study 2. Each cell shows the rank correlation between the model-derived genotype × isolate estimated marginal mean profiles of two isolates. In Study 1, each correlation was calculated using genotypes observed for both isolates in the pair (24 to 29 of the 30 genotypes); no value was imputed. In Study 2, all 12 genotypes were observed for every isolate. The colour scale ranges from −1 to 1. Correlations ranged from −0.04 to 0.82 in Study 1 (median 0.53) and from −0.62 to 0.66 in Study 2 (median 0.09); 16 of the 36 Study 2 correlations were negative. Cff089 was excluded because it was previously characterised as non-pathogenic on mungbean and did not provide an informative disease-severity profile for this analysis.

Table S6. Sensitivity of model tests and rankings to missing trifoliate scores.
The primary analysis used complete three-trifoliate assessments for the nine pathogenic isolates. Cff089 was excluded from all scenarios. The 126 records associated with failed germination or removal of late-germinating seedlings before inoculation were excluded from all scenarios. Only missing leaf scores in the 17 partially scored plants (four in Study 1; 13 in Study 2) were assigned 0 or 100 in the lower and upper scenarios, respectively; all recorded scores were retained. These hypothetical values were used only for sensitivity analysis.

A. Fixed-effect tests

Study	Scenario	Plants	Genotype	Isolate	Genotype × isolate	Year
1	Primary	957	43.999<br>(29, 684.66)<br>P <0.001	54.745<br>(8, 232.95)<br>P <0.001	4.842<br>(217, 552.87)<br>P <0.001	0.398<br>(1, 244.58)<br>P = 0.529
1	Lower	961	42.446<br>(29, 689.59)<br>P <0.001	54.220<br>(8, 233.57)<br>P <0.001	4.692<br>(217, 562.30)<br>P <0.001	0.634<br>(1, 243.83)<br>P = 0.427
1	Upper	961	43.991<br>(29, 689.02)<br>P <0.001	55.093<br>(8, 234.16)<br>P <0.001	4.820<br>(217, 561.07)<br>P <0.001	0.289<br>(1, 244.36)<br>P = 0.591
2	Primary	412	14.756<br>(11, 263.38)<br>P <0.001	30.142<br>(8, 98.94)<br>P <0.001	9.151<br>(88, 262.09)<br>P <0.001	0.545<br>(1, 104.51)<br>P = 0.462
2	Lower	425	14.998<br>(11, 275.68)<br>P <0.001	31.267<br>(8, 103.86)<br>P <0.001	9.026<br>(88, 274.61)<br>P <0.001	0.058<br>(1, 106.32)<br>P = 0.810
2	Upper	425	11.167<br>(11, 272.85)<br>P <0.001	20.048<br>(8, 103.73)<br>P <0.001	6.890<br>(88, 271.57)<br>P <0.001	5.476<br>(1, 106.27)<br>P = 0.021

Cells show F, followed by numerator and denominator degrees of freedom in parentheses, and P. Models retained genotype, isolate, their interaction and year as fixed effects and a pot random intercept. Kenward–Roger inference was used; main effects used flat-weighted EMM joint tests and the interaction used the full estimable interaction space. The Study 1 interaction has 217 degrees of freedom in all three analyses; the 15 previously unobserved genotype × isolate cells remain unobserved. Study 2 has 88 interaction degrees of freedom throughout.

B. Rank agreement with the primary analysis

Study	Scenario	Genotype ρ	Maximum genotype rank shift	Pathogenic isolate ρ	Maximum isolate rank shift
1	Lower	0.9996	1	1.0000	0
1	Upper	0.9996	1	1.0000	0
2	Lower	0.9930	1	1.0000	0
2	Upper	0.9720	2	0.9500	2

Ranks were calculated from flat-weighted marginal EMMs. Genotype comparisons include all 30 or 12 genotypes in Studies 1 and 2, respectively; isolate comparisons include the nine pathogenic isolates, excluding Cff089. ρ, Spearman rank correlation. Maximum shifts refer to individual ranks. The checks preserve significance of the three focal effects under the two specified scenarios, but do not establish that all rankings are insensitive to missing data or that exclusion bias is absent.

Table S7. Disease severity associated with the nine pathogenic *Curtobacterium flaccumfaciens* pv. *flaccumfaciens* (Cff) isolates in each study. Values are isolate estimated marginal mean (EMM) disease severity scores on the 0–300 scale, averaged across the observed genotype × isolate combinations and the two trials. The studies used different genotype sets and shared no genotype. Isolate EMMs therefore should not be compared directly between studies. Ranks are shown to describe changes in the relative ordering of isolates between studies. Isolates are listed in order of Study 1 disease severity.

Isolate	Genetic cluster[a]	Study 1 EMM	Study 1 95% CI	Study 1 Tukey[b]	Study 1 rank	Study 2 EMM	Study 2 95% CI	Study 2 Tukey[b]	Study 2 rank
Cff130	1	198.2	187.2 to 209.3	g	1	101.1	92.1 to 110.2	b	8
Cff062	2	196.3	185.4 to 207.2	fg	2	145.1	136.1 to 154.1	c	2
CffDA274	2	180.7	169.4 to 191.9	efg	3	143.7	134.6 to 152.8	c	3
CffT14089	4	171.2	159.7 to 182.7	ef	4	71.0	61.5 to 80.5	a	9
Cff021	6	156.3	145.0 to 167.6	de	5	117.9	108.8 to 127.0	b	4
CffT13799B	5	142.2	131.2 to 153.2	cd	6	106.1	96.7 to 115.4	b	7
DAR 56672	1	119.6	108.5 to 130.7	bc	7	152.6	143.5 to 161.7	c	1
DAR 58706	5	98.9	87.0 to 110.8	ab	8	115.6	106.3 to 124.9	b	5
DAR 56677	3	81.9	70.8 to 92.9	a	9	112.6	103.2 to 122.1	b	6

Note. [a] Cluster in the six-cluster population structure of Sparks et al. [6]. Full isolate metadata, including published plasmid pCff119 copy numbers, are given in Table 2. [b] Within a study, isolates sharing a letter did not differ at $\alpha = 0.05$ by Tukey's honestly significant difference procedure. Tukey letters are not comparable between studies. EMM, estimated marginal mean disease severity score; 95% CI, 95% confidence interval; Rank, position within that study from highest to lowest disease severity. Study 1 included 30 genotypes and Study 2 included 12.
